# Electrostatically Directed Self-Assembly of Ultrathin Supramolecular Polymer Microcapsules

**DOI:** 10.1002/adfm.201501079

**Published:** 2015-05-26

**Authors:** Richard M Parker, Jing Zhang, Yu Zheng, Roger J Coulston, Clive A Smith, Andrew R Salmon, Ziyi Yu, Oren A Scherman, Chris Abell

**Affiliations:** 1 Department of Chemistry, University of CambridgeLensfield Road, Cambridge, CB2 1EW, UK E-mail: ca26@cam.ac.uk; 2 Melville Laboratory for Polymer Synthesis, Department of Chemistry, University of CambridgeLensfield Road, Cambridge, CB2 1EW, UK; 3 Sphere Fluidics Limited, The Jonas Webb Building, Babraham Research Campus BabrahamCambridge, CB22 3AT, UK

**Keywords:** microcapsules, microfluidics, microstructures, self-assembly, supramolecular materials

## Abstract

Supramolecular self-assembly offers routes to challenging architectures on the molecular and macroscopic scale. Coupled with microfluidics it has been used to make microcapsules—where a 2D sheet is shaped in 3D, encapsulating the volume within. In this paper, a versatile methodology to direct the accumulation of capsule-forming components to the droplet interface using electrostatic interactions is described. In this approach, charged copolymers are selectively partitioned to the microdroplet interface by a complementary charged surfactant for subsequent supramolecular cross-linking via cucurbit[8]uril. This dynamic assembly process is employed to selectively form both hollow, ultrathin microcapsules and solid microparticles from a single solution. The ability to dictate the distribution of a mixture of charged copolymers within the microdroplet, as demonstrated by the single-step fabrication of distinct core–shell microcapsules, gives access to a new generation of innovative self-assembled constructs.

## 1. Introduction

The field of microencapsulation is rapidly expanding, with applications as diverse as cell encapsulation,[1] drug delivery,[2] catalysis,[3,4] food additives,[5] and incorporation within electronic displays.[6,7] Numerous approaches to microencapsulation have been reported, ranging from self-assembled polymersomes[8,9] and vesicles[10] to colloidosomes[11] and polymer microcapsules.[12–18] Synthetic routes to polymeric microcapsules typically rely on sacrificial core templates,[12] polyelectrolyte interactions,[13] cross-linked polymersomes,[19] covalent ­reactions,[14] supramolecular metal–organic films,[20] or hydrogen bonding.[15] These approaches often require multiple fabrication steps (e.g., layer-by-layer deposition or removal of a sacrificial template) and can be experimentally laborious.[21,22] Further, postfabrication loading of cargo, as often necessary with solid templates, considerably lowers encapsulation efficiency. In the absence of a well-defined template, microcapsules fabricated by bulk emulsification techniques often exhibit high polydispersity.[23]

The microemulsion of immiscible fluids offers an alternative route to hollow microcapsules, with the interfacial polymerization of segregated monomer solutions widely employed.[24,25] Here, loading of cargo can be achieved during fabrication, while disassembly triggers such as photocleavable groups can be incorporated into the monomer units to allow for on-demand, triggered release.[26] Further, interfacial aggregation has been successfully demonstrated to form self-repairing membranes at water/water interfaces, both via the aggregation of charged inorganic clusters (driven by cation exchange)[27] and the hierarchical self-assembly of small molecules on contact with an oppositely charged polymer solution.[28] More recently, layer-by-layer deposition has also been applied to liquid templates, such as oil-in-water microemulsions.[29,30] Although this multistep process remains time and labor intensive, developments in spray-drying are seeking to resolve this.[31]

As an emerging and powerful approach in microemulsification, microfluidic droplets offer a route to monodisperse microcapsules with identical composition.[29,32,33] In our previous work, we reported a one-step microfluidic approach to fabricate nanoparticle-embedded supramolecular microcapsules.[34] These dynamic yet highly stable capsules can encapsulate a cargo during formation with high loading and encapsulation efficiencies (>98%). Furthermore, the capsule wall can be controllably degraded through disruption of the supramolecular cross-links, allowing for triggered release of the cargo. As initially reported, microcapsule assembly was directed by the propensity of the gold nanoparticles to assemble at an oil–water droplet interface to form a Pickering emulsion, leading to accumulation of capsule-forming materials and ultimately the formation of a flexible supramolecular nanocomposite skin. Supramolecular cross-linking employed the macrocyclic host, cucurbit[8]uril (CB[8])[35] due to its ability to form stable yet dynamic 1:1:1 ternary charge-transfer complexes with polymer-bound electron-rich and electron-deficient guests, through ­multiple noncovalent interactions.[36] Their extremely high affinity in water is reported to be driven by the release of high energy water from the CB[8] cavity.[37]

A key step in the development of this approach is to generalize microcapsule fabrication away from the need to incorporate nanoparticles. This then requires an alternative method to control the accumulation of capsule-forming components at the periphery of the microdroplet. Herein, we present the assembly of supramolecular microcapsules from aqueous microdroplets driven by electrostatic interactions, whereby charged copolymers are selectively accumulated at the microdroplet interface by a complementary charged surfactant for subsequent supramolecular assembly via CB[8]. Electrostatic assembly is dynamic and reversible, with the location of components within the droplet able to be externally manipulated through the carrier oil. This ability to manipulate the distribution of microdroplet components is then employed in the selective formation of both hollow, ultrathin microcapsules and solid hydrogel microparticles from a single aqueous solution. Finally, the orthogonality of this electrostatic approach is demonstrated with the partitioning of a mixed solution of oppositely charged copolymers within the microdroplet, resulting in core–shell microcapsules upon evaporation.

## 2. Results and Discussion

**Figure 1:**
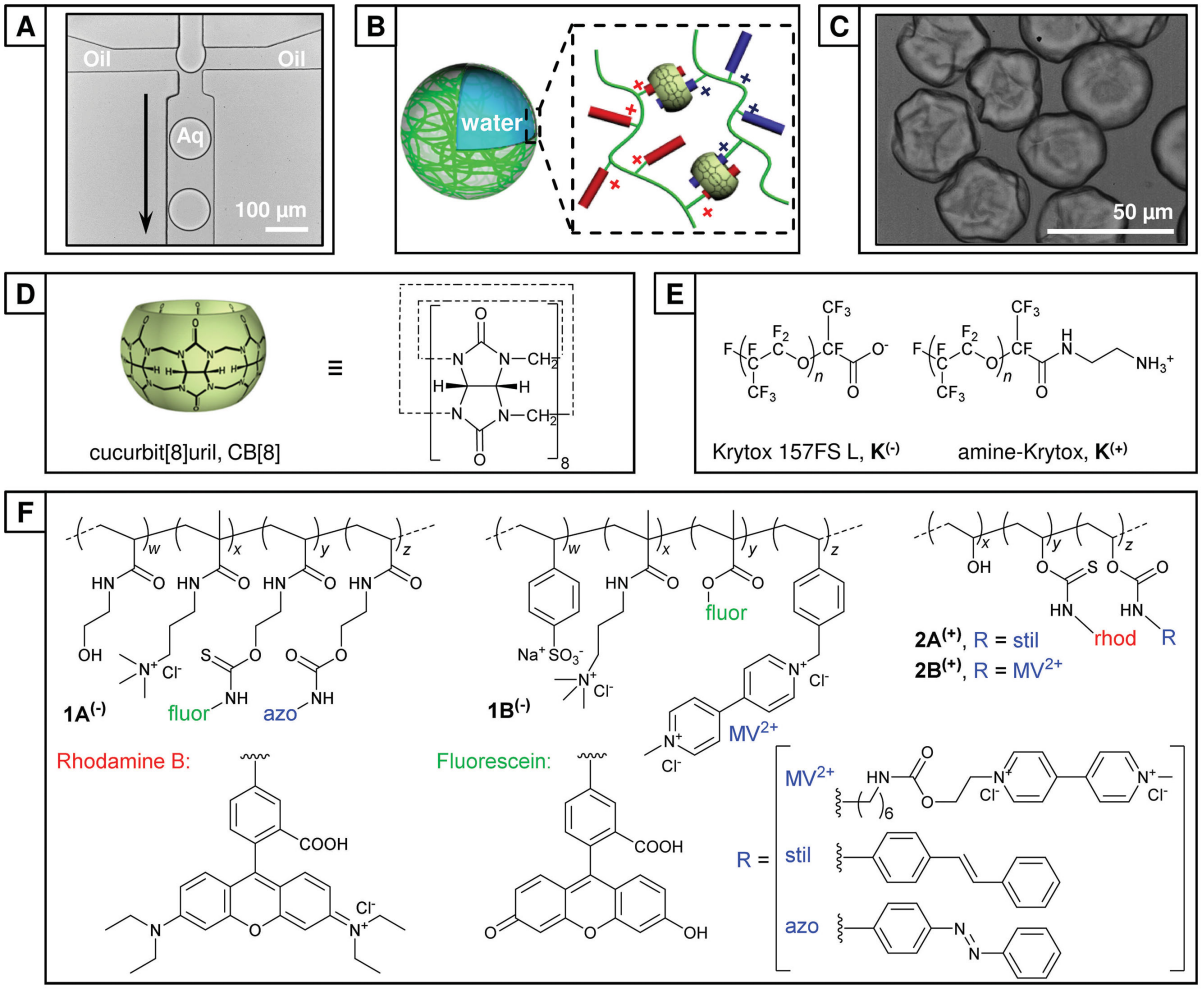
Formation of microcapsules from microfluidic droplets. A) Transmission optical image of the generation of monodisperse water-in-oil microdroplets (Ø = 79.0 ± 0.7 μm) at a 60 μm microfluidic flow-focusing junction. B) A schematic of supramolecular microcapsule formation between charged copolymers at the interface of a microdroplet. C) Transmission image of partially collapsed, ultrathin polymer microcapsules. D) Schematic and chemical structure of the cucurbit[8]uril macrocycle (CB[8]). E) Chemical structures of charged dopants: carboxylate-terminated Krytox (K^(−)^) and ammonium-terminated Krytox (K^(+)^). F) Chemical structures of the complimentary-functionalized copolymers 1A^(−)^ and 1B^(−)^ (net negative charge) and 2A^(+)^ and 2B^(+)^ (net positive charge).

Aqueous microdroplets were generated in a single step as an aqueous emulsion in oil within a polydimethylsiloxane (PDMS) microfluidic device, consisting of two inlets meeting at a single flow-focusing junction (60 × 50 μm, **Figure** **1**A and Figure S1A, Supporting Information). The continuous phase was composed from Fluorinert FC-40 perfluorinated oil (3M) containing 2.0 wt% fluorous surfactant (XL-01-171, Sphere Fluidics). To this was added up to 1.0 wt% of either a carboxylate-terminated (Krytox 157FS-L, DuPont, **K^(−)^**) or amine-terminated (**K^(+)^**) poly(hexafluoropropylene oxide) dopant. Once assembled at the water/oil boundary, these dopants give rise to either a negatively charged or positively charged interface, respectively. The dispersed phase consisted of an aqueous solution of charged, functionalized copolymer(s), and where appropriate a molar equivalent of CB[8]:guests. The low concentration of copolymer present within the stock solution prevents supramolecular gelation upon addition of an equivalent of CB[8] (60 × 10^−6^ m). For this work two pairs of oppositely charged copolymers were employed; each pair was fluorescently labeled and functionalized with complementary guest moieties for inclusion within CB[8]. The negatively charged copolymers poly(HEAm-*co*-AmAm-*co*-fluor-*co*-azo) (**1A^(−)^**) and poly(AmAm-*co*-SS-*co*-fluor-*co*-StMV) (**1B^(−)^**) were labeled with fluorescein (ex: 488 nm, em: 500–535 nm). Positively charged polymers poly(VA-*co*-rhodB-*co*-stil) (**2A^(+)^**) and poly(VA-*co*-rhodB-co-MV) (**2B^(+)^**) were labeled with rhodamine B (ex: 543 nm, em: 565–595 nm). The aqueous solution was injected at a flow rate of 75 μL h^−1^, where it intersected with a perpendicular continuous oil flow (150 μL h^−1^) and segmented into monodisperse microdroplets with a ­diameter (Ø) of 79.0 ± 0.7 μm (Figure S2, Supporting Information) and at a frequency of 80 Hz.

**Figure 2:**
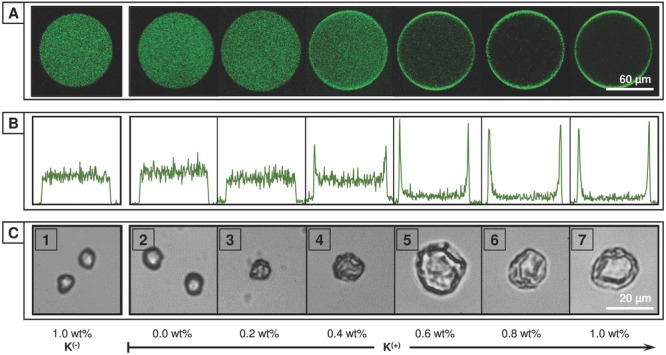
Electrostatically directed self-assembly of charged copolymers. A) Laser-scanning confocal fluorescent images and B) fluorescence intensity profiles of microdroplets containing an aqueous solution of fluorescein-labeled, negatively charged copolymer 1A^(−)^, [azo] = 60 × 10^−6^ m. Upon increasing the concentration of the positively charged Krytox, K^(+)^ within the carrier oil from 0.0 to 1.0 wt% the complementary charged copolymer is observed to accumulate at the droplet interface, templating microcapsule formation. C) Transmission images of evaporated microdroplets containing copolymers 1A^(−)^, 1B^(−)^, and CB[8] [azo:MV:CB[8] = 60:60:60 × 10^−6^ m]. As the concentration of K^(+)^ increases the morphology transitions from smooth, solid microparticles (1–3) to hollow, collapsed microcapsules (5–7).

Microdroplets comprising the negatively charged copolymer, **1A^(−)^** were prepared at 60 × 10^−6^ m concentration of azobenzene guest (**1A^(−)^** = 118 μg mL^−1^). During droplet generation the concentration of the charged dopant **K^(+)^** within the continuous phase was increased from 0.0 to 1.0 wt% and for comparison in the presence of 1.0 wt% **K^(−)^**. The microdroplets were collected into a storage reservoir (Figure S1B, Supporting Information) and the distribution of the fluorescent copolymer within the microdroplet immediately analyzed by laser-scanning confocal microscopy (LSCM). As shown in **Figure** **2**A,B, when a neutral or similarly charged surfactant (i.e., **K^(−)^**) is employed, **1A^(−)^** remains uniformly distributed throughout the microdroplet. However, upon increasing the concentration of the complementary charged **K^(+)^** the proportion of copolymer accumulated at the interface increases, with near-quantitative assembly above 0.4 wt% **K^(+)^**. A similar trend is observed for **1B^(−)^** (Figure S3, Supporting Information).

The distribution of copolymer within the microdroplet directly correlates with the resultant microstructure upon evaporation. Microdroplets containing a uniform distribution of copolymer throughout the droplet yield a solid, smooth microparticle upon evaporation, with only the successful partitioning of capsule-forming components to the droplet interface leading to the formation of a microcapsule. This is shown in Figure 2C, where microdroplets containing negatively charged copolymers **1A^(−)^** and **1B^(−)^** were prepared at 60 × 10^−6^m guest concentration (118 and 318 μg mL^−1^, respectively) and in a 1:1:1 guest ratio with CB[8] (80 μg mL^−1^). Upon evaporation of microdroplets with a uniform distribution of copolymer there is an observed decrease in droplet diameter until the concentration of copolymer eventually exceeds the solubility limit, leading to formation of a hydrogel microparticle (Figure 2C[1–3] and Figure S4B, Supporting Information). In contrast, when the microdroplet contents have been partitioned to the droplet interface a different regime is observed. Once the microdroplet has undergone a sufficient degree of evaporation (Ø ≈ 70% Ø*_t_*_= 0_ at 60 × 10^−6^ m), the concentration of CB[8] at the interface rises to initiate significant supramolecular cross-linking of the preorganized copolymers (**1A^(−)^** ⊂ CB[8] ⊂ **1B^(−)^**). This results in the formation of a polymeric shell around the microdroplet, encapsulating its contents in a single step. Upon further evaporation, the aqueous droplet no longer fills the microcapsule shell and it collapses (Figure 1C and Figure S4A, Supporting Information), with marked folds and creases formed (Figure 2C[5–7]).

Microcapsule formation was similarly observed for positively charged copolymers **2A^(+)^** and **2B^(+)^** exclusively in the presence of the carboxylate-terminated Krytox, **K^(−)^** (Figure S6, Supporting Information); **2A^(+)^** and **2B^(+)^** were prepared at 60 × 10^−6^ m guest concentration (44 and 66 μg mL^−1^, respectively) and in a 1:1:1 guest ratio with CB[8] (80 μg mL^−1^). In the absence of CB[8], although partitioning within the microdroplet is unperturbed, only smooth, polymer microparticles are formed upon evaporation (Figure S7, Supporting Information). This control experiment confirms that supramolecular cross-linking between copolymer chains is crucial to undergo the droplet-to-capsule transition. Further, it should also be noted that microcapsule formation is not dependent on the ­composition of the oil phase, with both perfluorinated (FC-40) and organic oils (exemplified with hexadecane in Figure S13, Supporting Information) successfully employed.

**Figure 3:**
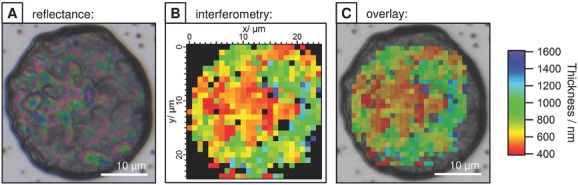
Interferometric mapping of microcapsule thickness. A) Reflectance image of a dried, collapsed microcapsule made from of 2A^(+)^, 2B^(+)^, and CB[8] [stil:MV:CB[8] = 60:60:60 × 10^−6^ m]. B) Thickness map of the microcapsule as determined by optical interferometry, with a 1.5 μm spot size and automated microscope movement, and C) overlaid.

The thickness of the dry microcapsule skin was determined by optical interferometry.[38] This technique exploits the optical interference resulting from the path length difference between reflections from the microcapsule–air and microcapsule–substrate interfaces upon exposure to polychromatic light (400–900 nm). The average single wall thickness for **1A^(−)^** ⊂ CB[8] ⊂**1B^(−)^** microcapsules was measured as 380 ± 50 nm, while microcapsules assembled from **2A^(+)^** ⊂ CB[8] ⊂ **2B^(+)^** were thinner—with a wall thickness of 170 ± 50 nm. The marked difference in wall thickness is due to the lower proportion of guest moieties on **1A^(−)^** and **1B^(−)^**, corresponding to a higher total material loading per droplet (130 pg/droplet) than for **2A^(+)^** ⊂ CB[8] ⊂**2B^(+)^** (50 pg/droplet) when standardized to 60 × 10^−6^ m guest concentration. This exemplifies that microcapsule wall thickness can be tailored by both the copolymer morphology and the absolute concentration within the microdroplet. By scanning the collection region over the microcapsule via an automated stage, the interference pattern and consequently the microcapsule thickness could be resolved spatially (**2A^(+)^** ⊂ CB[8] ⊂ **2B^(+)^**, **Figure** **3**). In the central region the “double wall” thickness was found to be 400–600 nm. Importantly, spatial mapping identifies the thickness variations in individual microcapsules arising from folds and creases, allowing interpretation of the variation in microcapsule thickness measurements.

The measured wall thickness is thicker than would typically be expected by an interfacial complexation mechanism, such as the electrostatic deposition of a polyelectrolyte layer (as employed in layer-by-layer assembly). However, it is proposed that the supramolecular microcapsules are only templated by the electrostatic interaction with an oppositely charged surfactant at the droplet interface. This interaction is responsible for the initial accumulation of charged copolymer as a thin, diffuse layer at the interface (as observed in Figure 2), with the copolymer acting similarly to a surfactant. With capsule formation shown to be an evaporative process, it is expected that this interfacial polymer layer dynamically reorganizes and reflows upon reduction in droplet surface area, while still held at the interface by the dynamic supramolecular cross-links. At a critical point this layer undergoes a phase change, locking the structure into a flexible polymeric film that buckles and collapses upon further evaporation.

**Figure 4:**
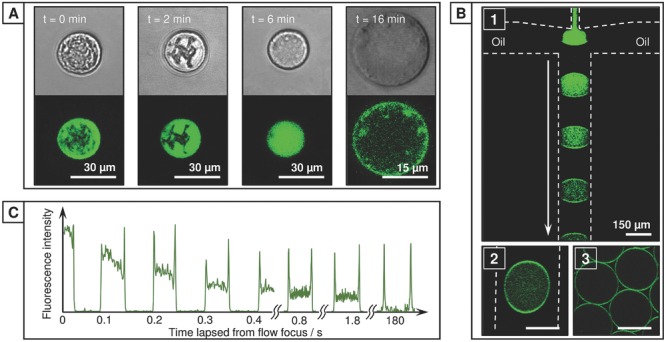
Microcapsule inflation and tracking copolymer accumulation within the microdroplet. A) Rehydration of 1A^(−)^ ⊂ CB[8] ⊂1B^(−)^ microcapsules containing dextran cargo (10 × 10^−6^m, 70 kDa) confirms that the polymeric skin is retained. B) Real-time laser-scanning confocal fluorescent images of microdroplets containing an aqueous solution of fluorescein-labeled, negatively charged copolymer 1A^(−)^, [azo] = 60 × 10^−6^ m. In the presence of K^(+)^ (1.0 wt%) diffusion of 1A^(−)^ to the droplet interface rapidly progresses as it flows along the microfluidic channel, from (1) flow focus to (2) the channel outlet; (3) after leaving the delivery tubing near-quantitative diffusion to the interface had occurred. Dashed lines mark channel boundaries. C) Fluorescence intensity profiles across a transect of a microdroplet as a function of the time-lapsed since generation at the flow-focusing junction, illustrating the kinetics of the rapid diffusion to the droplet interface.

The simultaneous delivery of the capsule-forming components and aqueous-soluble cargo as a single flow during microdroplet formation enables trivial encapsulation of cargo within the microcapsule. Here, microcapsules (**1A^(−)^** ⊂ CB[8] ⊂ **1B^(−)^**, 60 × 10^−6^ m) were assembled containing 10 × 10^−6^ m of dextran (70 kDa) as a macromolecular cargo. While this encapsulated dextran did not interfere with the partitioning of **1A^(−)^** and **1B^(−)^** to the interface and corresponding capsule formation, it enables the collapsed microcapsule to be osmotically reinflated post-evaporation. Hydration of the microcapsule, as studied by LSCM (**Figure** **4**A), shows that as the capsule refills the creases and folds diminish, confirming that the top and lower faces of the capsule are still separate (i.e., the final stages of evaporation do not result in the formation of a microparticle disk). Despite being exclusively formed from soluble components, the strength of the CB[8] ternary complex (*K*_a_ up to 10^12^ m^−2^ in water)[39,40] endows high stability to the hydrated microcapsule, with no ­evidence of dissolution over 24 h. The retention of macro­molecular cargo (fluorescein-labeled dextran, 250 kDa, 6.7 × 10^−6^ m) within the microcapsule (**2A^(+)^** ⊂ CB[8] ⊂ **2B^(+)^**, 60 × 10^−6^ m) was confirmed by LSCM (Figure S8, Supporting Information); subsequent addition of a competitive guest (1-adamantylamine, 2 × 10^−3^ m) resulted in rapid dispersal of this cargo into bulk media. This ability to retain cargo within an ultrathin microcapsule wall demonstrates the high material loading efficiency (>98 wt%) of the supramolecular polymer microcapsules.

The accumulation of **1A^(−)^** at the droplet interface was studied by real-time LSCM using an expanded microfluidic exit and oil channels (200 × 50 μm) to slow the droplet flow velocity (droplet diameter, Ø = 194 μm, 5 Hz). As shown in Figure 4B,C, in the presence of 1.0 wt% **K^(+)^**, **1A^(−)^** will begin to diffuse (aided by chaotic advection) to the oil–water boundary upon droplet formation at the flow-focusing junction (B1). As the droplet travels along the microfluidic channel this diffusion process continues, with assembly at the interface near-complete after 1.8 s (3.6 mm, B2); on leaving the exit tubing (10 cm, 180 s) all copolymer had been partitioned (B3). The rate at which **1A^(−)^** is partitioned slows on decreasing the concentration of **K^(+)^** within the carrier oil (Figure S5, Supporting Information), with partitioning to the interface not observed for low concentrations (0–0.4 wt%) of **K^(+)^** or with **K^(−)^**. For positively charged copolymer **2B^(+)^**, rapid partitioning to the interface was similarly exclusively observed in the presence of **K^(−)^** (Figure S9, Supporting Information). While increasing the relative flow rate of the carrier oil effectively raises the dopant concentration per droplet—increasing the partitioning rate, altering physical parameters such as the droplet size or the droplet generation rate were found to have little effect. Here a linear channel was employed, however it is expected that geometries designed to increase mixing within the droplet (e.g., a winding channel) would increase the partitioning rate.

The ability to trigger capsule formation through evaporative concentration allows for the microarchitecture to be tailored postdroplet formation (Figure S4C, Supporting Information). For example, neutral droplets would tend to form microparticles, however the subsequent introduction of a complementary charged dopant to the oil phase will dynamically reorganize the copolymer to the microdroplet interface, leading to capsule formation. Conversely, dilution of the oil phase will reduce the effective concentration of any charged dopant, reversibly switching the droplet from a capsule-forming to particle-forming regime. If the dopant concentration is subsequently restored by either additional dopant or evaporative concentration of the oil phase, capsule formation can be reinstated. Significantly, the addition of a conflicting charged dopant will disperse the copolymer from the droplet interface, inhibiting capsule formation. It should be noted that once supramolecular cross-linking has progressed to form a skin, the microcapsule architecture is functionally fixed.

**Figure 5:**
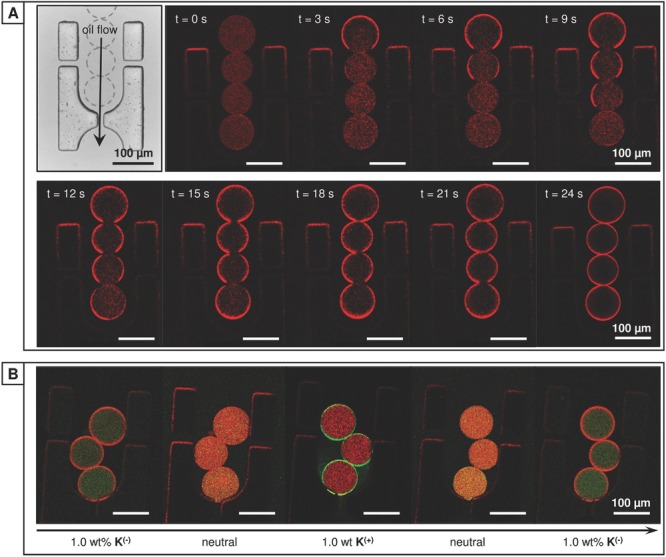
Dynamic control of charged copolymer within the microdroplet. A) Transmission image of the microdroplet trap (top left); aqueous microdroplets of rhodamine-labeled 2B^(+)^ [MV = 60 × 10^−6^ m] were held within the trap by a continuous oil flow (250 μL h^−1^). Real-time laser-scanning confocal fluorescent images of the trapped microdroplets within a neutral oil flow. Upon switching to a complimentarily charged surfactant (1.0 wt% K^(−)^) rapid diffusion to the droplet interface was observed, with partitioning complete in 24 s. B) The location of orthogonally charged copolymers 1A^(−)^ and 2B^(+)^ [azo:MV = 60:60 × 10^−6^ m] can be independently manipulated within the microdroplet, allowing for selective assembly at the interface.

To investigate the kinetics of electrostatically driven assembly of charged copolymers, microdroplets containing **2B^(+)^** were prepared at 60 × 10^−6^ m concentration of viologen guest (66 μg mL^−1^). The droplets were confined within microfluidic “traps” (Figure S1C, Supporting Information) under positive pressure by a continuous flow of oil (250 μL h^−1^). As shown in **Figure** **5**A, under neutral conditions **2B^(+)^** was initially uniformly distributed throughout the microdroplet. On introduction of 1 wt% of **K^(−)^** to the continuous oil flow, **2B^(+)^** was immediately drawn to the interface of the upstream droplet, with each successive droplet switching in turn as the dopant accumulated at the interface. This transition from dispersed to interfacial assembly was observed to take 24 s at this flow rate (<3 s for an isolated droplet), with the sheltered interdroplet regions the last section to switch. Interestingly assembly is sufficiently dynamic that despite the lag between switching at the upstream and downstream hemispheres, **2B^(+)^** is still uniformly distributed around the microdroplet. This accumulation of **2B^(+)^** can be reversed over the same timescale by reverting to a neutral flow of oil. Further, no loss of efficacy or material was observed over repeated cycling between **K^(−)^** and **K^(+)^** (Figure S10, Supporting Information).

To explore the orthogonality of electrostatic assembly, microdroplets containing both negatively charged **1A^(−)^** and positively charged **2B^(+)^** at 60 × 10^−6^ m guest concentration were trapped. As shown in Figure 5B, upon flowing an oil containing the positively or negatively charged dopant the complementary charged copolymer was exclusively drawn to the oil/water interface. On returning to a neutral oil flow, the copolymer at the interface is observed to again disperse throughout the droplet. Further, introducing an equimolar quantity of CB[8] did not alter the ability or rate at which the distribution of the complementary functionalized copolymers **1A^(−)^** and **2B^(+)^** could be manipulated within the droplet. As with the single copolymer system, this dynamic process is both repeatable and reproducible.

**Figure 6:**
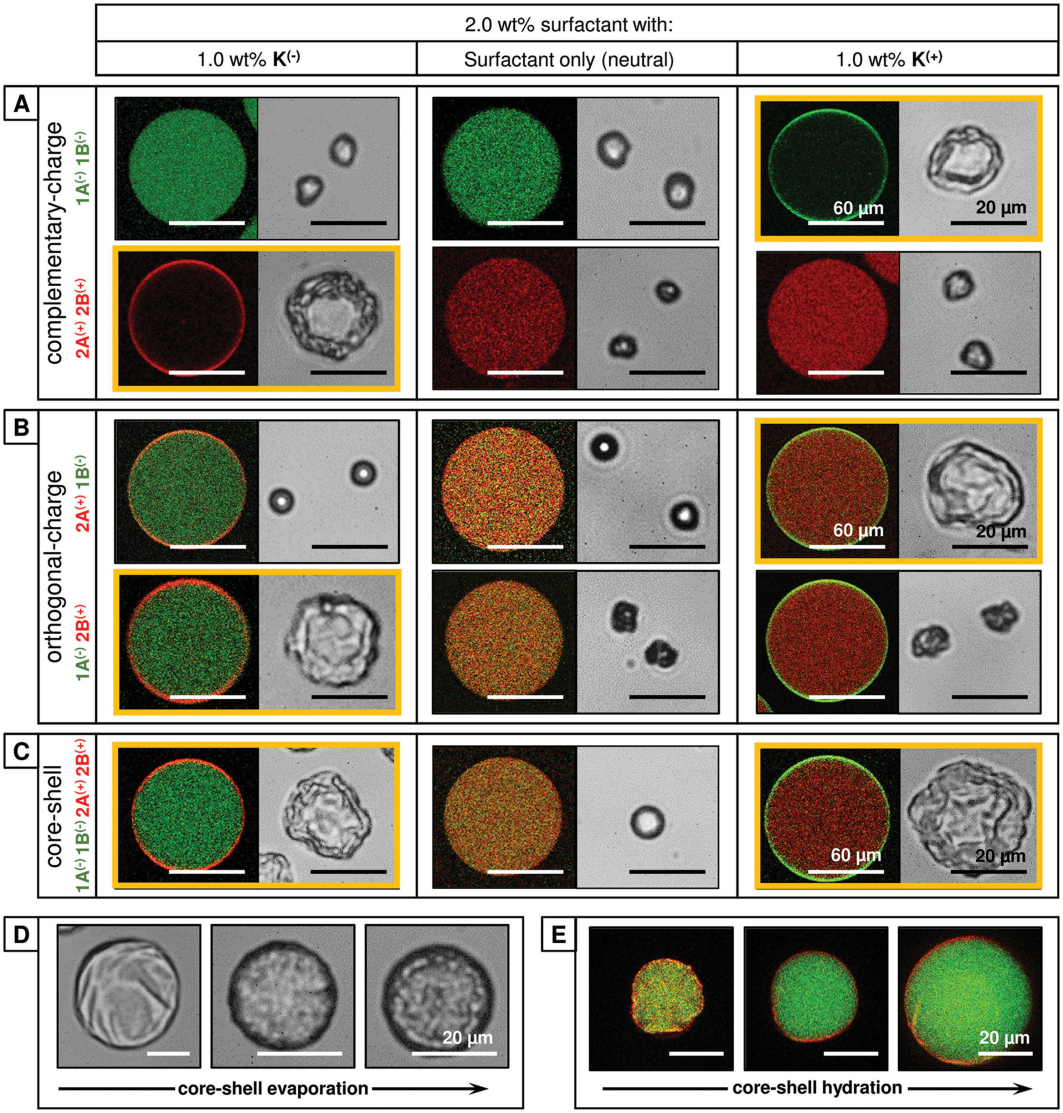
Table correlating the distribution of fluorescent copolymer in the microdroplet (left) with the resultant microstructure formed upon evaporation (right), as a function of the charge at the droplet interface. A) Complementary: Negative copolymers 1A^(−)^ and 1B^(−)^ (fluorescein-labeled, green) were found to exclusively form a hollow microcapsule in the presence of K^(+)^ [azo:MV:CB[8] = 60:60:60 × 10^−6^ m]. Conversely, positive copolymers 2A^(+)^ and 2B^(+)^ (rhodamine-labeled, red) were found to exclusively form a microcapsule with K^(−)^ [stil:MV:CB[8] = 60:60:60 × 10^−6^ m]. B) Orthogonal: A mixed solution of negative 1A^(−)^ and positive 2B^(+)^ was found to exclusively form a hollow microcapsule in the presence of K^(−)^ [azo:MV:CB[8] = 60:60:60 × 10^−6^ m]. Conversely, a mixed solution of positive 2A^(+)^ and negative 1B^(−)^ exclusively formed microcapsules with K^(+)^ [stil:MV:CB[8] = 60:60:60 × 10^−6^ m]. C) *Core–shell*: A mixed solution of 1A^(−)^, 1B^(−)^, 2A^(+)^, and 2B^(+)^ formed homogenous microparticles under neutral conditions. However, core–shell microcapsules were formed with both K^(+)^ and K^(−)^ dopants, with the outer shell comprising of either 1A^(−)^ ⊂ CB[8] ⊂ 1B^(−)^, or 2A^(+)^ ⊂ CB[8] ⊂ 2B^(+)^, respectively [CB[8]:guests = 120:60 × 10^−6^ m]. D) Transmission images of the evaporative formation of a core–shell microcapsule containing 160 × 10^−6^ m of dextran cargo (70 kDa) and E) laser-scanning confocal fluorescence image of this core–shell microcapsule during hydration at *t* = 0, 15, and 30 min, illustrating the partitioned copolymers within the cross-linked microstructure (shell: 2A^(+)^ ⊂ CB[8] ⊂ 2B^(+)^, core: 1A^(−)^ ⊂ CB[8] ⊂ 1B^(−)^).

The ability to template the resultant microarchitecture by manipulation of the microdroplet offers an unprecedented level of control to dynamically tailor the design to the application. This concept is illustrated in **Figure** **6**A, where the distribution of fluorescent copolymers in the microdroplet is correlated with the microstructure formed upon evaporation. The assembly of systems where both copolymers respond equally to the applied charge is simple to predict; in the presence of CB[8], negative copolymers **1A^(−)^** and **1B^(−)^** (fluorescein-labeled, green) were found to exclusively form a hollow microcapsule in the presence of **K^(+)^**, while conversely negative copolymers **2A^(+)^** and **2B^(+)^** (rhodamine-labeled, red) required **K^(−)^**. All disfavored interactions resulted in the formation of microparticles. In such binary systems there is only one possible supramolecular complex that can form with CB[8], allowing the microscale assembly to dominate the resultant microarchitecture.

When orthogonally charged copolymers **2A^(+)^** and **1B^(−)^** are employed in the presence of CB[8], only a single copolymer is held at the interface by either charged dopant, leading to segregation of copolymer within the microdroplet. While it may be expected that the segregation of copolymers would inhibit supramolecular cross-linking, microcapsule formation is exclusively observed with **K^(+)^** (Figure 6B, top right). A similar trend is observed for the second pair of copolymers, **1A^(−)^** and **2B^(+)^**, however microcapsule formation is now exclusively observed with **K^(−)^** (Figure 6B, bottom left). This dichotomy, where microcapsule formation is not only possible, but occurs only when a specific copolymer from the pair is drawn to the interface can be explained through the supramolecular assembly process. In both examples of successful microcapsule formation, it is the viologen-functionalized copolymer (**1B^(−)^**, **2B^(+)^**) that is driving assembly at the interface. CB[8] is known to bind strongly to methyl viologen (*K*_a_ = 10^5^ m^−1^),[41] with the resulting MV ⊂ CB[8] complex capable of subsequently binding an electron-rich aromatic second guest in a second step (Figure S11, Supporting Information).[36] As such the assembly of orthogonally charged binary systems can still be predicted, however both the microscale and supramolecular self-assembly processes must be considered.

Binary mixtures of copolymers have been shown to controllably form either microparticles or microcapsules, however more complex architectures can be formed from mixtures of competing copolymers. Microdroplets were generated containing a mixed solution of **1A^(−)^**, **1B^(−)^**, **2A^(+)^**, **2B^(+)^** and CB[8] with [CB[8]:guests = 120:60 × 10^−6^m]. In this extended system the presence of orthogonally charged, viologen-functionalized copolymers **1B^(−)^** and **2B^(+)^** ensures that supramolecular assembly can independently occur both at the interface and throughout the microdroplet. In the presence of **K^(+)^**, copolymers were segregated by charge within the microdroplet to form core–shell microcapsules, where an outer **1A^(−)^** ⊂ CB[8] ⊂ **1B^(−)^** shell encapsulated a **2A^(+)^** ⊂ CB[8] ⊂ **2B^(+)^** hydrogel core (Figure 6C). Similarly, the inverted structure can be formed in the presence of **K^(−)^**. The evaporative formation of core–shell microcapsules occurred in two stages, with the ultrathin microcapsule skin forming first, with the residual copolymers remaining in solution within the microcapsule until the critical concentration for gelation was met (Figure 6D). This inner phase does not fill the microcapsule in the dehydrated state, allowing the shell to collapse. The collapsed, dry core–shell microcapsules were osmotically reinflated on hydration via a dextran cargo (70 kDa, 160 × 10^−6^ m), allowing the supra­molecular core–shell morphology to be confirmed (Figure 6E and Figure S12, Supporting Information).

## 3. Conclusion

By combining electrostatically directed self-assembly on the microscale with the precise molecular recognition offered by supramolecular chemistry, we describe a highly versatile and effective methodology to fabricate supramolecular polymer microcapsules. This approach exploits the electrostatic interaction between charged copolymers and charged surfactants (independent of molecular weight, specific polymer chemistry, or the composition of the hydrophobic phase) to selectively assemble capsule-forming components to the interface of a microdroplet, where they subsequently cross-link via a supramolecular ternary complex with CB[8]. By only utilizing electrostatics in structure direction (rather than microcapsule assembly) a low proportion of charged monomers (<1%) is required within the copolymer. The presence of charge along the copolymer backbone does not interfere with host–guest chemistry,[40] allowing for structure direction and copolymer assembly to be controlled synergistically, but independently. The wide range of potential host–guest interactions available offers a direct route to on-demand release of cargo by a range of stimuli,[34,42–44] however this methodology is anticipated to also extend to covalently assembled systems.

In contrast to other routes to microcapsule preparation, supramolecular microcapsules were formed in a single-step from a single-emulsion microdroplet, with all cargo and capsule-forming components introduced in a single fluid flow. This not only allows for the precise quantity and ratio of each component within the microcapsule to be controlled, but for high material loading and encapsulation efficiencies, with all components isolated from the external environment from the point of droplet formation; this is a distinct advantage over interfacial assembly of supramolecular microcapsules.[45] It should however be stressed that electrostatically directed assembly is not reliant on microfluidics, with supramolecular microcapsules readily formed at the interface of even simple microemulsions of two complementary charged phases. While recent droplet(s)-in-droplet based emulsion strategies have enabled the synergistic encapsulation of cargoes or the use of miscible fluids,[9,18] the considerable interfacial tension between different phases and requirement for complex microfluidics limits their versatility. More importantly, the general reliance on covalent cross-linking to form the microcapsule structure limits the pathways available for on-demand release of cargo.

The ability to dictate the distribution of multiple charged polymers within the microdroplet, templating the resultant microstructure, gives access to a wide range of self-assembly constructs on demand. This concept was demonstrated by the single-step fabrication of distinct core–shell microcapsules, however by manipulation of the microdroplet template (e.g., Janus droplets or multiple emulsions) more complicated architectures are projected. This will give ready access to compartmentalized structures, where each constituent capsule could be tailored in terms of permeability or release trigger. This will enable new applications, ranging from the synergistic storage and selective release of divergent cargos, to investigating the kinetics of (bio)chemical processes in a controlled, localized environment.[46]

## 4. Experimental Section

*Materials*: Commercially available compounds and solvents were obtained from Sigma-Aldrich or Alfa Aesar and used without further purification, unless noted. Cucurbit[8]uril was synthesized as previously reported (see the Supporting Information). Charged surfactants were provided by Sphere Fluidics. All aqueous solutions were prepared in deionized water (Millipore Milli-Q Gradient A10) ensuring a resistivity of >15 MΩ cm^−1^. Full synthetic methods and characterization for copolymers and charged surfactants are included in the Supporting Information.

*1A^(−)^ and 1B^(−)^: Negatively Charged Copolymers*: Water-soluble polyacrylamide copolymers **1A^(−)^** and **1B^(−)^** employed fluorescein as a fluorescent label and to introduce negative charge, [fluor] = 1%. **1A^(−)^** contained 7% of the electron-rich guest, azobenzene (*M*_W_ = 121 kDa, *M*_n_ = 103 kDa, polydispersity index (PDI) = 1.2). **1B^(−)^** contained 4% of the electron-poor guest, methyl viologen with styrene sulfonate included to maintain a net negative charge (*M*_w_ = 34 kDa, *M*_n_ = 28 kDa, PDI = 1.2).

*2A^(+)^ and 2B^(+)^: Positively Charged Copolymers*: Water-soluble **2A^(+)^** and **2B^(+)^** were derived from polyvinylalcohol (Mowiol 6-98, *M*_W_ = 47 kDa) and functionalized with rhodamine B isocyanate to introduce both fluorescence and positive charge, [rhod] = 1%. **2A^(+)^** was further functionalized with the electron-rich guest, stilbene isocyanate (*M*_W_ = 73 kDa, *M*_n_ = 56 kDa, PDI = 1.3, [Stil] = 10%), while **2B^(+)^** was functionalized with the electron-poor guest, 6-(methyl viologen)hexamethylene isocyanate (*M*_W_ = 51 kDa, *M*_n_ = 39 kDa, PDI = 1.3, [MV] = 5%).

*Charged Surfactants, K^(−)^ and K^(+)^*: Commercial carboxylate terminated poly(hexafluoropropylene oxide) (Krytox 157FS-L DuPont) was used as the negative dopant, **K^(−)^**. The positive dopant, **K^(+)^**, was synthesized by coupling Krytox 157FS-L to ethylene diamine, via the acid chloride.

*Instrumentation*: Microdroplets were imaged using a Vision Research Phantom Miro ex4-M fast camera, attached to an Olympus IX71 inverted microscope (10×–64× objectives). Laser-scanning confocal microscope fluorescence measurements were carried out using a Leica TCS SP5 confocal microscope using a 10× objective. Fluorescent labels, fluorescein (ex: 488 nm, em: 500–535 nm) and rhodamine B (ex: 543 nm, em: 565–595 nm) were used to track the location of copolymers within the microdroplet.

*Droplet-Based Microfluidics*: Microfluidic devices were fabricated from PDMS via soft lithography (further details in the Supporting Information). To render the channels fluorophilic they were immediately flushed with a 0.5% v/v solution of trichloro(1H,1H,2H,2H-perfluorooctyl)silane in Fluorinert FC-40 (3M) and subsequently cured at 120 °C overnight. Device designs are shown in Figure S1 (Supporting Information). Monodisperse water-in-oil microdroplets were generated with a hydrophobic flow-focusing microfluidic channel (Figure S1A, Supporting Information). The diameter of the junction was 60 μm with a channel depth of 50 μm. To generate microdroplets, the continuous oil phase and the discrete aqueous phase were injected into the microfluidic device via two syringe pumps (PHD 2000, Harvard Apparatus) with controlled flow rates of 150 and 75 μL h^−1^, respectively. At the intersection, the shear forces caused the formation of aqueous droplets in oil (Ø = 79.0 ± 0.7 μm, Figure S2, Supporting Information). The continuous phase comprised of the perfluorinated oil, Fluorinert FC-40 (3M), with 2 wt% surfactant (XL-01-171, Sphere Fluidics). To this was added up to 1.0 wt% of either the carboxylate (**K^(−)^**) or amine-terminated (**K^(+)^**) poly(hexafluoropropylene oxide) dopant. The dispersed phase consisted of an aqueous solution of charged, functionalized copolymer(s), and where appropriate a molar equivalent of CB[8]:guests. For a typical experiment, a concentration of 60 × 10^−6^ m was used for each polymer-bound guest and CB[8] to allow formation of the ternary 1:1:1 complex.

*Optical Interferometry*:[38] Microcapsule-wall thickness was spectrally determined from the optical interference pattern arising from the path length (and thus, phase) difference between reflections from the upper microcapsule–air and lower microcapsule–glass interfaces. Consequently, the intensity of the resulting reflection will oscillate as a function of wavelength. It can be derived that the film thickness, *t*, is given by Equation (1)


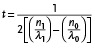
(1)

where *n*_1_ and *n*_0_ are the refractive indices of the polymeric microcapsule skin at the wavelengths *λ*_1_ and *λ*_0_, respectively, which correspond to neighboring intensity minima in the spectrum. Reflection spectra were taken from microscopic samples by coupling a spectrometer (Ocean Optics QE65000) to a modified commercial microscope (Olympus BX51) though a 50 μm core optical fiber in a conjugate focal plane. The tungsten–halogen lamp of the microscope (spectral range: 400–900 nm) was used as the light source and spectra were normalized by the reflection from the glass substrate. Spectral data were collected from the center of 65 microcapsules and automatically analyzed using custom software; the quoted error represents both the uncertainty in the refractive index of the microcapsule film and the sample-to-sample variation due to creases and folds, with the latter dominating. For thickness mapping, automatic stage movement was used to collect spectra from a spatial grid of areas. The data were collected in the form of a hyperspectral cube, with two spatial dimensions and a third spectral dimension. The thickness map consists of a thickness value for each spatial pixel, determined from its associated spectra. In the central regions where there are little folds or creases, the measured “double-wall” thickness is consistent with the above measurements.

## Supporting Information

Supporting Information is available from the Wiley Online Library or from the author. Additional data related to this publication is available at the University of Cambridge data repository (https://www.repository.cam.ac.uk/handle/1810/247727).

As a service to our authors and readers, this journal provides supporting information supplied by the authors. Such materials are peer reviewed and may be re-organized for online delivery, but are not copy-edited or typeset. Technical support issues arising from supporting information (other than missing files) should be addressed to the authors.
