# Supplementary material for: Electrostatically Directed Self-Assembly of Ultrathin Supramolecular Polymer Microcapsules
Source: Adv Funct Mater. 2015 May 26;25(26):4091–100. doi: 10.1002/adfm.201501079 (PMC4511391; doi:10.1002/adfm.201501079)
Supplement: Supplementary file 1 — Supplementary [file adfm0025-4091-sd1.pdf]

Copyright WILEY-VCH Verlag GmbH & Co. KGaA, 69469 Weinheim, Germany, 2015.

ADVANCED FUNCTIONAL MATERIALS

# Supporting Information

for *Adv. Funct. Mater.,* DOI: 10.1002/adfm.201501079

# Electrostatically Directed Self-Assembly of Ultrathin Supramolecular Polymer Microcapsules

*Richard M. Parker, Jing Zhang, Yu Zheng, Roger J. Coulston, Clive A. Smith, Andrew R. Salmon, Ziyi Yu, Oren A. Scherman, and Chris Abell**

## Electronic Supporting Information

# Electrostatically-directed Self-assembly of Ultra-thin Supramolecular Polymer Microcapsules

Richard M. Parker,[1] Jing Zhang,[1] Yu Zheng,[2] Roger J. Coulston,[2] Clive A. Smith,[3] Andrew R. Salmon,[1] Ziyi Yu,[1] Oren A. Scherman[1,2] and Chris Abell[1]*

E-mail: ca26@cam.ac.uk (C.A.); oas23@cam.ac.uk (O.A.S.)

[1] Dr R. M. Parker, Dr J. Zhang, A. R. Salmon, Dr Z. Yu, Prof C. Abell
Department of Chemistry, University of Cambridge, Lensfield Road, Cambridge CB2 1EW, UK

[2] Dr Y. Zheng, Dr R. J. Coulston, Dr O. A. Scherman
Melville Laboratory for Polymer Synthesis, Department of Chemistry, University of Cambridge, Lensfield Road, Cambridge CB2 1EW, UK

[3] Dr C. A. Smith
Sphere Fluidics Limited, The Jonas Webb Building, Babraham Research Campus, Babraham, Cambridge CB22 3AT, UK

## S1: Microfluidic device design and microdroplet generation

Microfluidic devices were manufactured from polydimethylsiloxane (PDMS) *via* soft lithography, whereby: (i) the microchannel network was designed *in silico (AutoCAD)*, (ii) printed as a negative photo-mask and (iii) transferred onto a silicon wafer spin-coated with SU-8 photoresist *via* UV-photolithography to form a mould. PDMS and the cross linker (Sylgard 184 elastomer kit, *Dow Corning*) in a 10:1 ratio were poured onto this mould and allowed to stand overnight at 70 °C. The PDMS layer, imprinted with the microfluidic channel design, was removed and using a biopsy punch (1.0 mm) inlets and an outlet were formed. The imprinted PDMS and a glass substrate were exposed to oxygen plasma for 8 s and then pressed together to seal the microfluidic channels.

To render these channels hydrophobic, they were immediately flushed with a 0.5% *v/v* solution of trichloro(1H,1H,2H,2H-perfluorooctyl)silane in Fluorinert FC-40 (*3M*) and subsequently cured at 120 °C overnight. Device designs are shown in Figure S1.

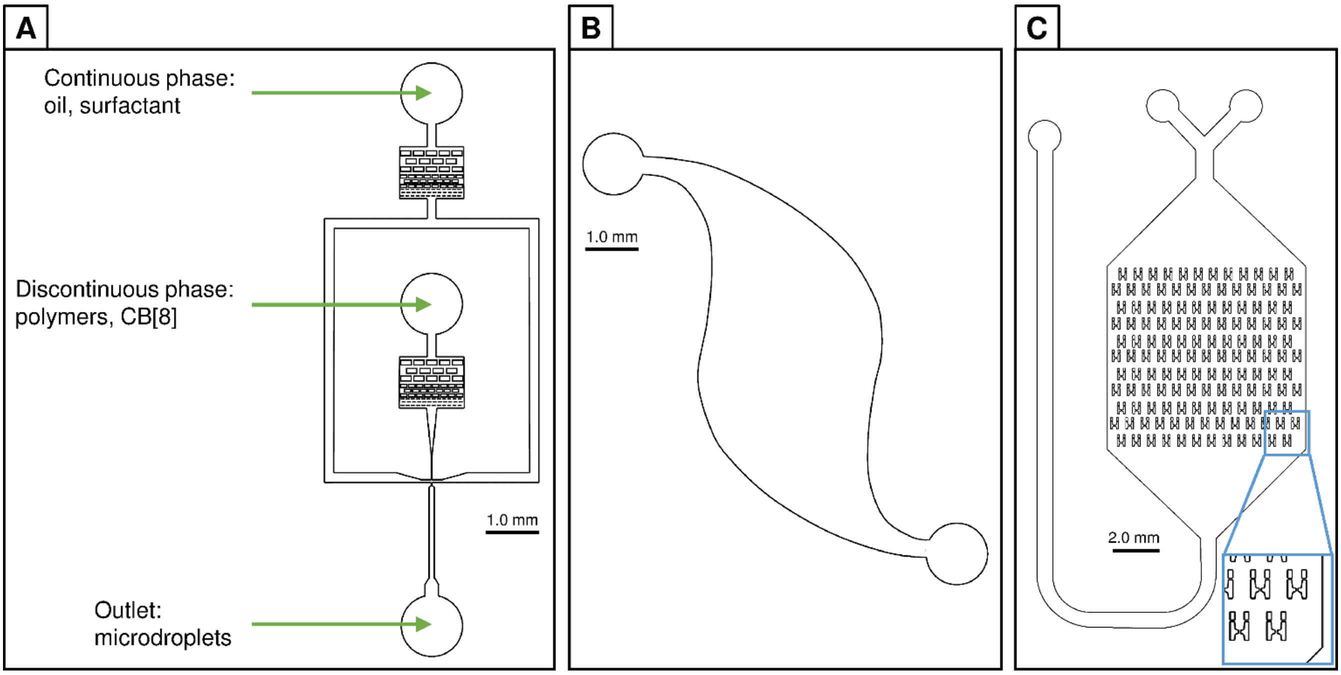


**Figure S1 | Schematic of microfluidic devices.** (**A**) Water-in-oil microdroplets were generated using a hydrophobic microfluidic device comprising a 60 µm flow-focusing nozzle. (**B**) Analysis of static microdroplets was conducted in a microfluidic reservoir. (**C**) A droplet-trapping reservoir was employed when a continuous oil flow was required; the droplet-trapping pillars are shown in the inset. All microfluidic channels were 50 µm in depth.

Monodisperse water-in-oil microdroplets were generated with a hydrophobic flow-focusing microfluidic channel (Figure S1A). The diameter of the junction was 60 µm with a channel depth of 50 µm. To generate microdroplets, the continuous oil phase and the discrete aqueous phase were injected into the microfluidic device *via* two syringe pumps (PHD 2000, *Harvard Apparatus*) with controlled flow rates of 150 and 75 $\mu Lh^{-1}$ respectively. At the intersection, the shear forces caused the formation of aqueous droplets in oil (Ø = 79.0 ± 0.7 µm, Figure S2). The continuous phase comprised of the perfluorinated oil, Fluorinert FC-40 (*3M*), with 2 wt% surfactant (XL-01-171, *Sphere Fluidics*). To this was added up to 1.0 wt% of either the carboxylic acid ($\mathbf{K^{(-)}}$) or amine-terminated ($\mathbf{K^{(+)}}$) poly(hexafluoropropylene oxide) dopant. The dispersed phase consisted of an aqueous solution of charged, functionalized copolymer(s), and where appropriate a molar equivalent of CB[8]:guests. For a typical experiment, a concentration of 60 µM was used for each polymer-bound guest and CB[8] to allow formation of the ternary 1:1:1 complex.

Once formed, microdroplets were collected either into a microfluidic reservoir (Figure S1B,C) or on the surface of a glass slide for further study and evaporative formation of microcapsules. Residual surfactant was removed by washing with solvent (FC-40), upon which it can be recycled.

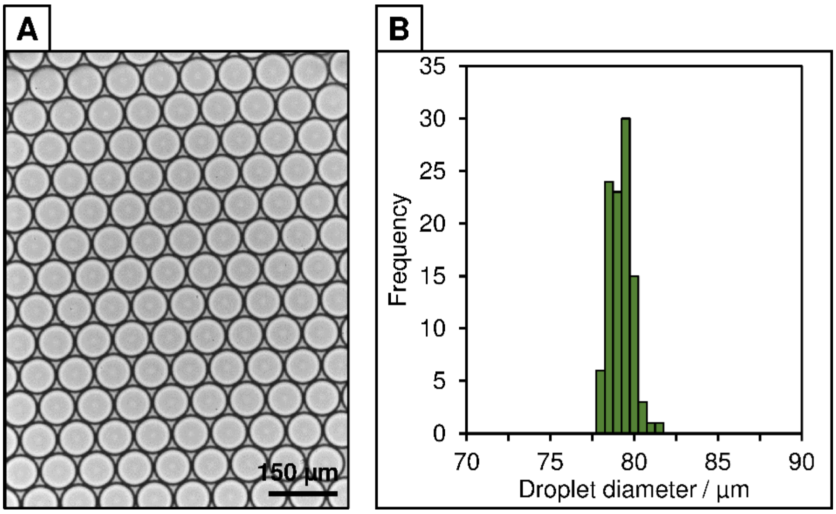


**Figure S2 | Monodisperse aqueous microdroplets.** (**A**) Transmission optical micrograph of monodisperse aqueous microdroplets (Ø = 79.0 ± 0.7 µm) and (**B**) the corresponding histogram of droplet diameters, exhibiting the narrow size distribution (coefficient of variation 0.8%).

## S2: ‘1A$^{(-)}$⊂CB[8]⊂1B$^{(-)}$’ Microcapsules

Microdroplets comprising the negatively-charged copolymer, **1A**$^{(-)}$ were prepared at 60 µM concentration of azobenzene guest. During droplet generation the concentration of the charged-dopant **K**$^{(+)}$ within the continuous phase was increased from 0.0 to 1.0 wt% and for comparison in the presence of 1.0 wt% **K**$^{(-)}$. The microdroplets were collected into a storage reservoir (Figure S1B) and the distribution of the fluorescent copolymer within the microdroplet immediately analyzed by laser-scanning confocal microscopy (LSCM). As shown in Figure S3A, when a neutral surfactant is employed **1A**$^{(-)}$ remains uniformly distributed throughout the microdroplet. However upon increasing the concentration of the complementarily-charged **K**$^{(+)}$ the proportion of copolymer accumulated at the interface increases, with near-quantitative assembly at 0.6 wt% **K**$^{(+)}$. A similar trend is observed for **1B**$^{(-)}$, with significant accumulation at the interface observed with just 0.4 wt% **K**$^{(+)}$ (Figure S3B). A mixed solution of **1A**$^{(-)}$ and **1B**$^{(-)}$, in the presence of CB[8], follows the same trend (Figure S3C), however doubling the total copolymer concentration reduces the effect of the charged-dopant.

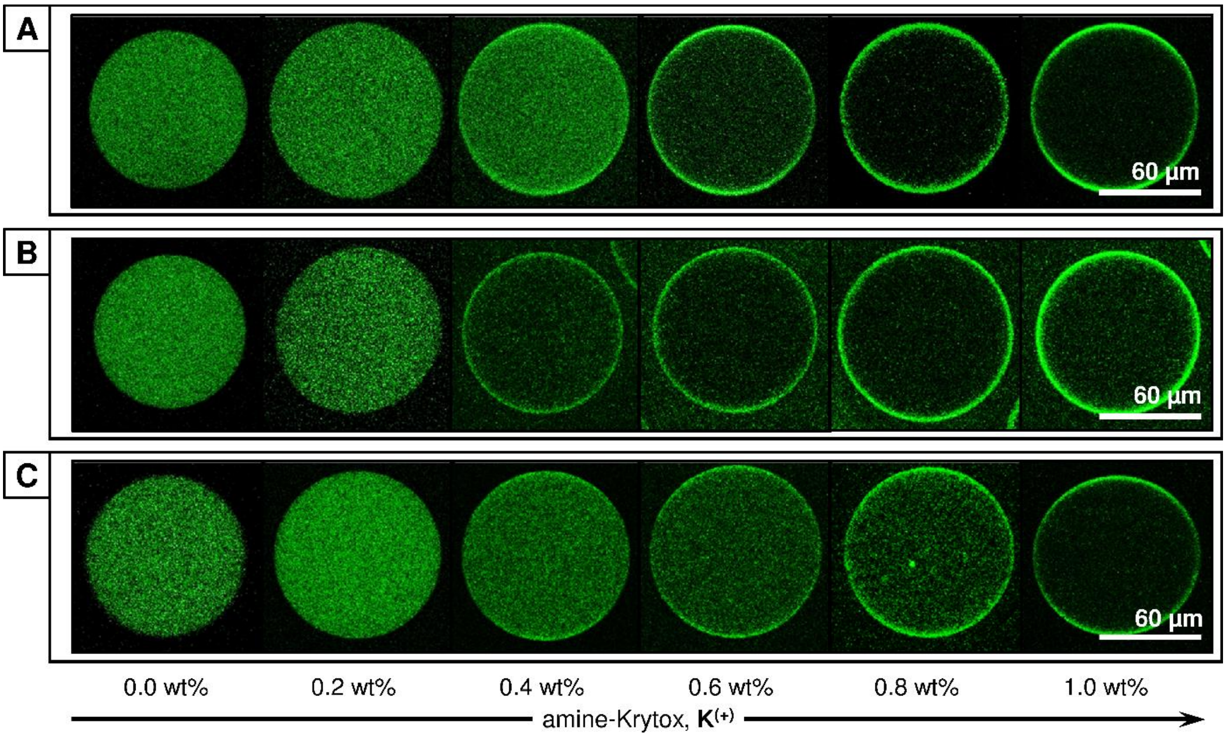


**Figure S3 | Controlled assembly of charged copolymers.** LSCM of microdroplets containing an aqueous solution of fluorescein-labelled, negatively-charged copolymer(s): (**A**) **1A**$^{(-)}$**,** [azo] = 60 µM; (**B**) **1B**$^{(-)}$ [$MV^{2+}$] = 60 µM; and (**C**) **1A**$^{(-)}$, **1B**$^{(-)}$ and CB[8], [azo : MV : CB[8] = 60 : 60 : 60 µM]. Upon increasing the concentration of **K**$^{(+)}$ within the carrier oil from 0.0 wt% to 1.0 wt% the complementary charged copolymers are observed to accumulate at the droplet interface, templating microcapsule formation.

Any significant accumulation of copolymer at the interface on evaporation will typically lead to the formation of hollow microcapsules (Figure S4A), while microdroplets containing dispersed copolymer will form solid microparticles (Figure S4B). The hollow microcapsule forms creases and folds as it collapses, contrasting strongly with the smooth, dense microparticle.

The ability to trigger capsule formation through evaporative concentration allows for the micro-architecture to be tailored post-droplet formation (Figure S4C). Methods for dynamically switching the location of charged copolymers within the droplet include: changes in dopant concentration (e.g. evaporation or dilution of solvent, addition of dopant) or the addition of a conflicting charged-dopant. It should be noted that once supramolecular cross-linking has progressed sufficiently to form a microcapsule skin or hydrogel, the micro-architecture is fixed.

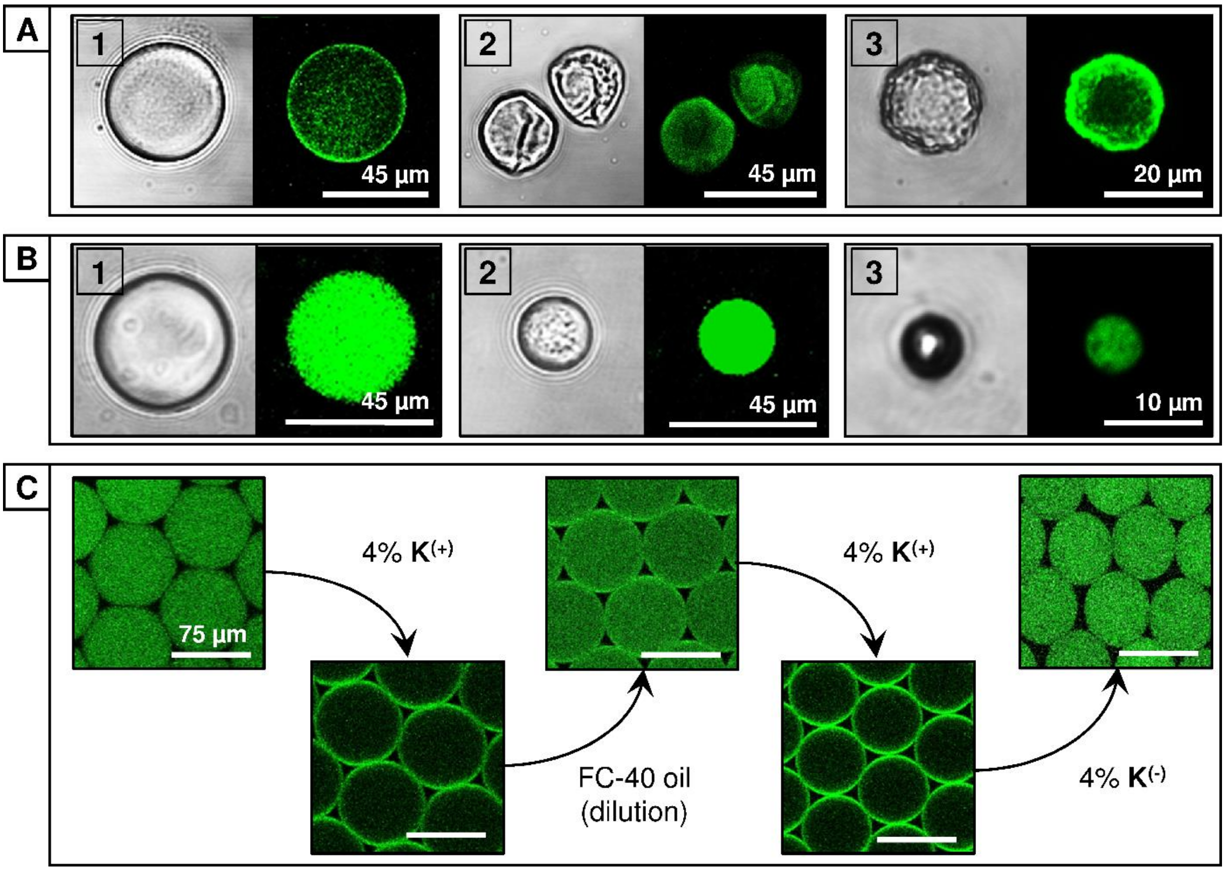


**Figure S4 | 1A$^{(-)}$⊂CB[8]⊂1B$^{(-)}$ microcapsule formation.** Transmission and LSCM of microcapsules formed from copolymers **1A$^{(-)}$**, **1B$^{(-)}$** and CB[8], [azo : MV : CB[8] = 60 : 60 : 60 µM]. (**A**) Evaporation of the microdroplet in the presence of 1.0 wt% **K$^{(+)}$** leads to the formation of a ultra-thin polymeric skin (A1); upon further evaporation this hollow structure is observed to wrinkle (A2) and collapse (A3). (**B**) In the absence of **K$^{(+)}$**, copolymers do not accumulate at the droplet interface, resulting in a solid microparticle upon evaporation (B1-3). (**C**) LSCM micrographs illustrating that dynamic manipulation of the copolymer within the microdroplet can be achieved through the addition of charged-dopants or carrier oil.

The accumulation of **1A**$^{(-)}$ at the droplet interface was studied by real-time LSCM using expanded microfluidic exit and oil channels (200 x 50 µm) to slow the droplet flow velocity. Figure S5 plots the ratio of the fluorescence intensity at the droplet interface against the bulk volume of the droplet, as the microdroplet flows along the microfluidic channel. In the presence of **K**$^{(+)}$, **1A**$^{(-)}$ will begin to move to the oil-water boundary upon droplet formation at the flow-focusing junction, with this process continuing until fluorescence is only observed at the interface. The rate at which **1A**$^{(-)}$ is partitioned slows on lowering the concentration of **K**$^{(+)}$ within the carrier oil , with partitioning not observed for low or neutral concentrations (≤ 0.4 wt% **K**$^{(+)}$).

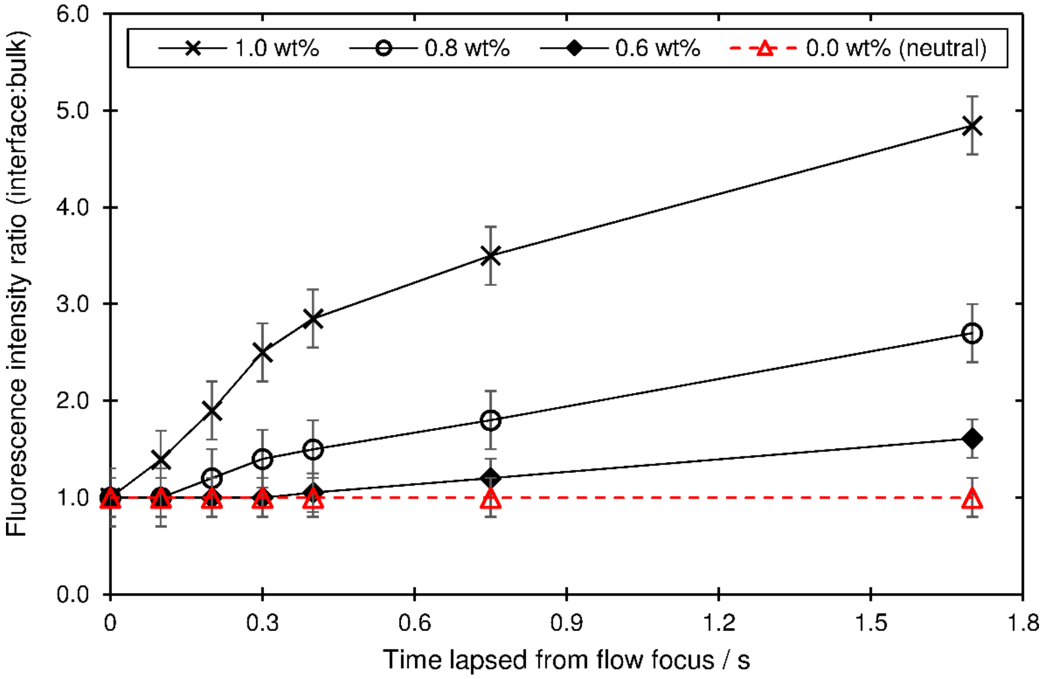


**Figure S5 | Temporal study of the assembly of 1A$^{(-)}$ at the droplet interface.** The ratio of the fluorescence intensity at the droplet interface against that within the bulk volume of the microdroplet is plotted as a function of time lapsed from droplet generation at the flow focus. The rate of movement to the droplet interface increases with increasing the concentration of complementary-charged surfactant, **K**$^{(+)}$, in contrast no accumulation is observed under neutral conditions (red). Lines are added to guide the eye.

## S3: '2A$^{(+)}$⊂CB[8]⊂2B$^{(+)}$' Microcapsules

Microdroplets containing **2A**$^{(+)}$ and **2B**$^{(+)}$ can be directed to form microcapsules or microparticles exclusively with **K**$^{(-)}$, as shown in Figure S6. When microcapsules were formed, LSCM studies showed that copolymers were retained at the interface throughout the evaporative process (Figure S6B). Fast-drying microcapsules tend to collapse into compact structures, while slower drying microcapsules tend to flatten, giving rise to the dispersity of shapes observed.

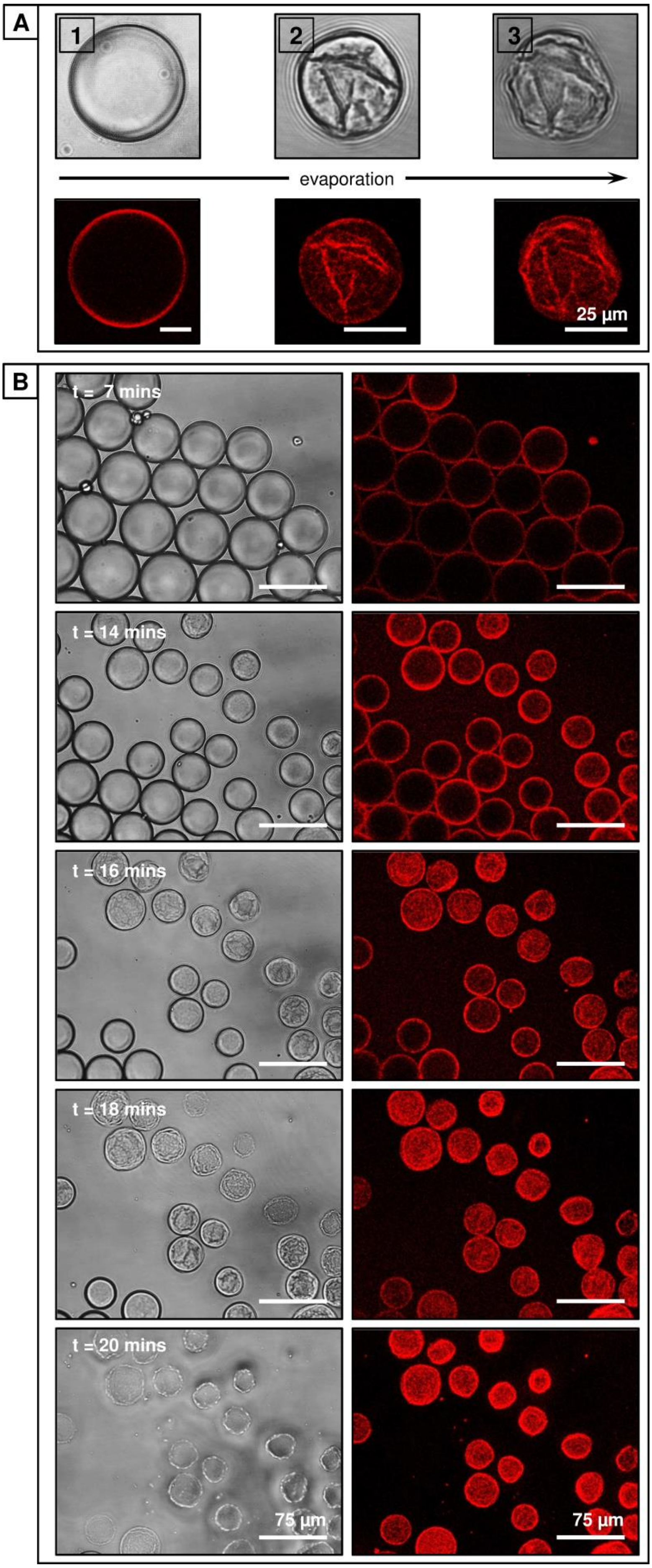


**Figure S6 | 2A$^{(+)}$⊂CB[8]⊂2B$^{(+)}$ microcapsule formation.** Transmission and LSCM micrographs of aqueous microdroplets of rhodamine-labelled **2A**$^{(+)}$, **2B**$^{(+)}$ and CB[8] [stil : MV : CB[8] = 60 : 60 : 60 µM] during microcapsule formation. (**A**) Droplet evaporation: (i) in the presence of 1.0 wt% **K**$^{(-)}$ copolymers **2A**$^{(+)}$, **2B**$^{(+)}$ accumulate at the droplet interface, (ii) supramolecular cross-linking leads to the formation of skin at the interface that upon further evaporation is no longer filled by the aqueous droplet leading to collapse and (iii) dry. (**B**) Time-resolved micrographs of the evaporation driven transition from microdroplets to microcapsules, as directed by the pre-accumulation of copolymers **2A**$^{(+)}$ and **2B**$^{(+)}$ at the droplet interface.

In the absence of CB[8], although partitioning within the microdroplet is unperturbed, only smooth, polymer microparticles are formed upon evaporation. This control experiment confirms that supramolecular cross-linking between copolymer chains is crucial to undergo the microcapsule formation (Figure S7).

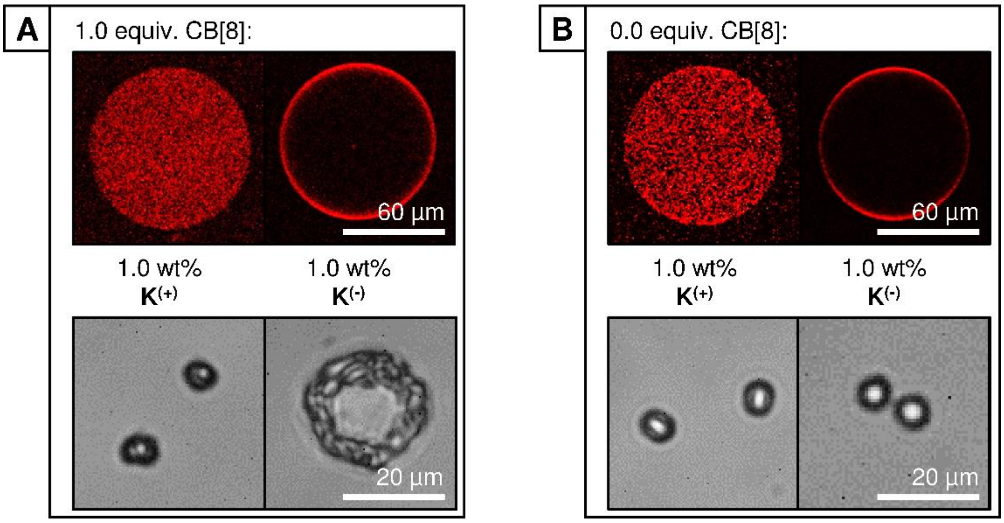


**Figure S7 | Confirmation of the role of supramolecular cross-linking in microcapsule formation.** LSCM micrographs of aqueous microdroplets of rhodamine-labelled **2A**$^{(+)}$ and **2B**$^{(+)}$ [stil : MV = 60 : 60 µM] (top) and transmission micrographs of the corresponding polymeric microstructure upon evaporation (bottom). (**A**) In the presence of 1.0 equiv. CB[8], the microdroplet templates the resultant microstructure, giving rise to either irregular microparticles or hollow microcapsules. (**B**) In the absence of CB[8] smooth microparticles are formed irrespective of the distribution of copolymer within the microdroplet.

Hydrophilic cargo can be simultaneously loaded within the microdroplet, allowing one-step encapsulation with high efficiency. Fluorescein-labelled dextran (250 kDa) was used as a model cargo, allowing its location to be tracked by LSCM. Osmotic rehydration of the microcapsule, as shown in Figure S8B, confirms the cargo (green) is retained within the thin polymer shell (red). In contrast, microparticles are smaller, with the cargo homogeneously mixed throughout the polymer network (Figure S8C).

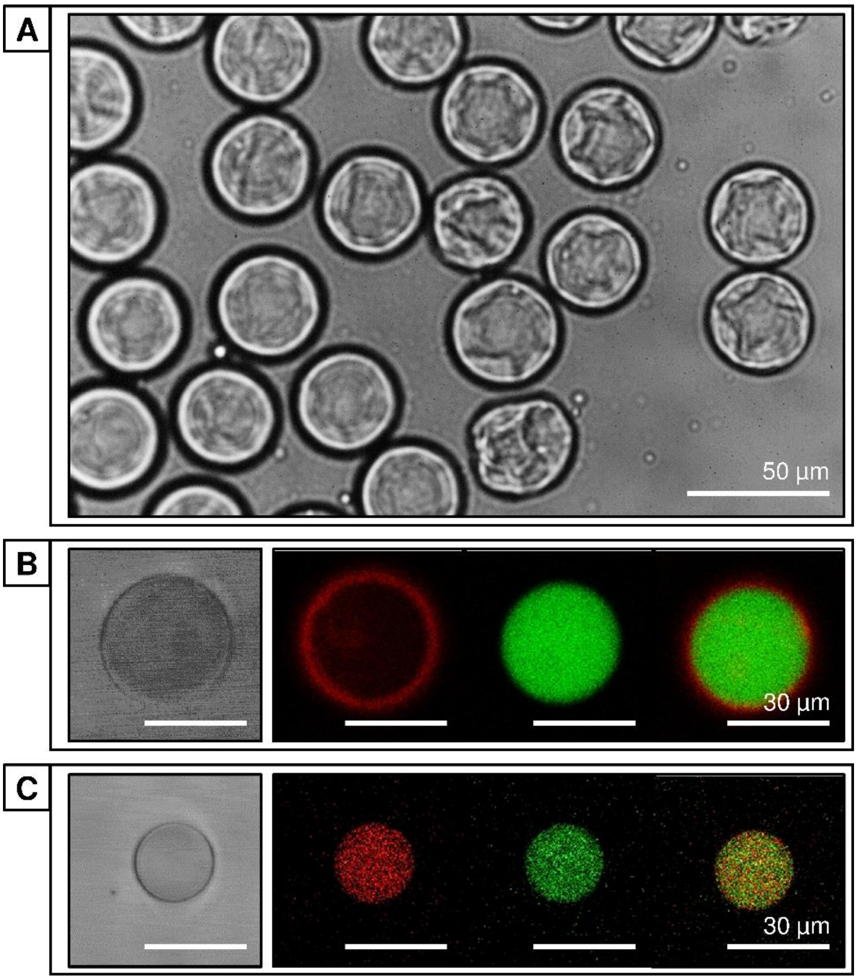


**Figure S8 | Hydration of cargo-loaded microcapsule.** Transmission micrograph of partially collapsed microcapsules containing rhodamine-labelled **2A**$^{(+)}$, **2B**$^{(+)}$ and CB[8] [stil : MV : CB[8] = 60 : 60 : 60 µM], containing a fluorescein-dextran cargo (6.7 µM, 250 kDa). (**B,C**) Transmission and LSCM micrographs of hydrated microstructures: (**B**) hydration of a microcapsule gives rise to a distinct hollow 'skin' (red) encapsulating the macromolecular cargo (green); in contrast, a hydrated microparticle (**C**) has a homogenous internal structure.

The accumulation of positively-charged **2B**$^{(+)}$ at the droplet interface was studied by real-time LSCM using expanded microfluidic exit and oil channels (200x50 µm) to slow the droplet flow velocity (droplet diameter = 194 µm, 5 Hz). In the presence of **K**$^{(-)}$, rapid partitioning to the interface was observed (Figure S9). In the absence of **K**$^{(-)}$ the driving force is removed, with **2B**$^{(+)}$ remaining dispersed throughout the droplet.

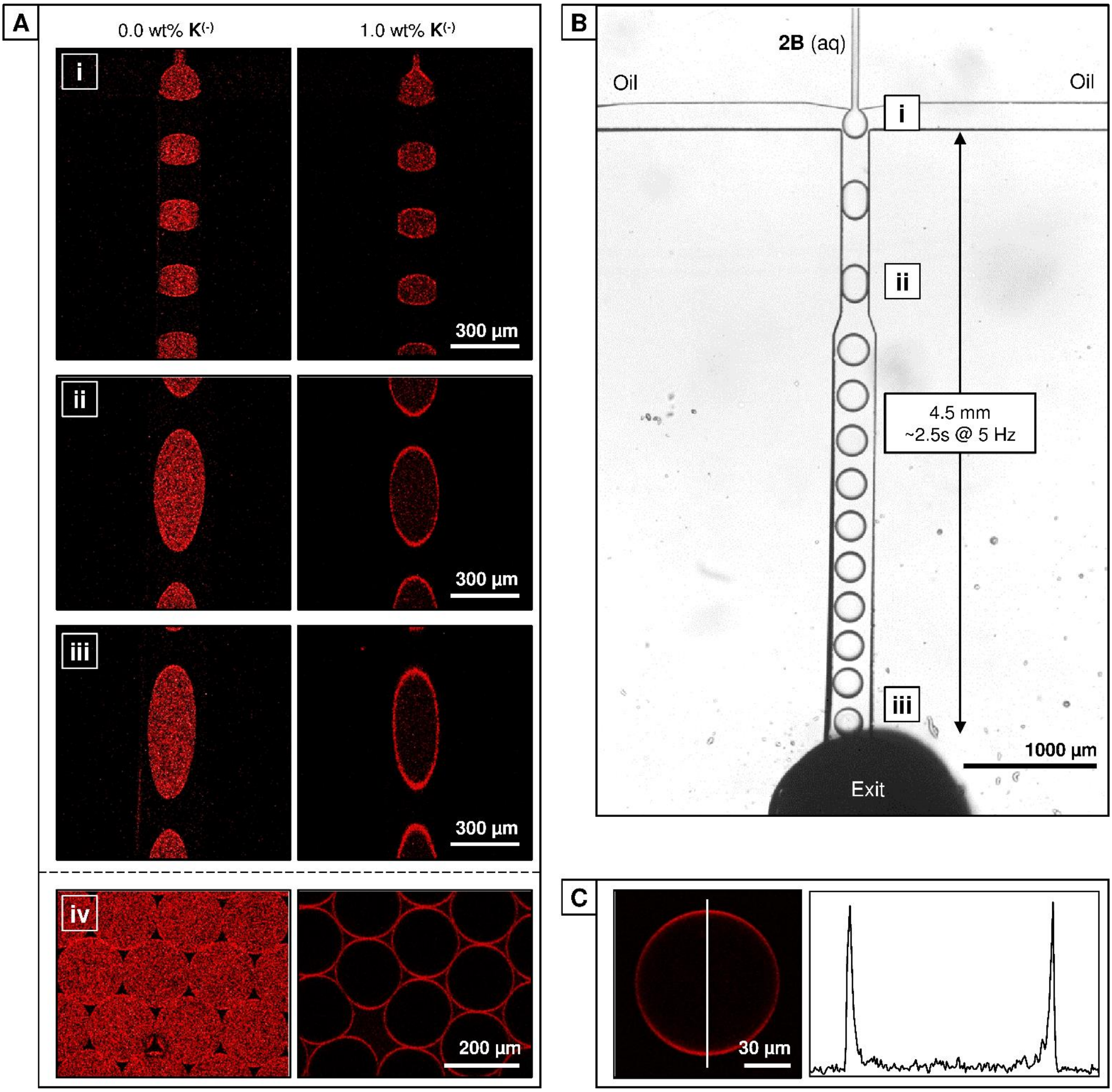


**Figure S9 | Assembly of 2B$^{(+)}$ at the microdroplet interface.** LSCM micrographs of microdroplets containing rhodamine-labelled **2B**$^{(+)}$ [MV = 60 µM]. (**A**) In the presence of the complementarily-charged **K**$^{(-)}$ (1.0 wt%), diffusion of **2B**$^{(+)}$ to the droplet interface rapidly progresses as it flows along the microfluidic channel, from (i) flow focus to (iii) the channel exit; (iv) after leaving the delivery tubing near-quantitative diffusion to the interface had occurred. In the absence of **K**$^{(-)}$ no preference for the interface is observed. (**B**) Transmission micrograph of the enlarged flow-focusing device (200x50 µm), illustrating the measurement locations (i-iii). (**C**) Fluorescence intensity profile along a transect of a single microdroplet, illustrating the near-quantitative assembly of **2B**$^{(+)}$ at the droplet interface.

To investigate the kinetics of electrostatic-driven assembly of charged copolymers, microdroplets containing positively-charged **2B**$^{(+)}$ were prepared at 60 µM concentration of viologen guest and confined within microfluidic 'traps' (Figure S1C) under positive pressure by a continuous flow of oil (250 µLh$^{-1}$). On introduction of 1 wt% of **K**$^{(-)}$ to the continuous oil flow, **2B**$^{(+)}$ was immediately drawn to the interface of the upstream droplet. This accumulation of **2B**$^{(+)}$ can be reversed over the same timescale by reverting to a flow of 1 wt% **K**$^{(+)}$. Repeated cycling between **K**$^{(-)}$ and **K**$^{(+)}$ leads to alternating between diffuse and interfacial assembly, with no loss of efficacy or material observed (Figure S10).

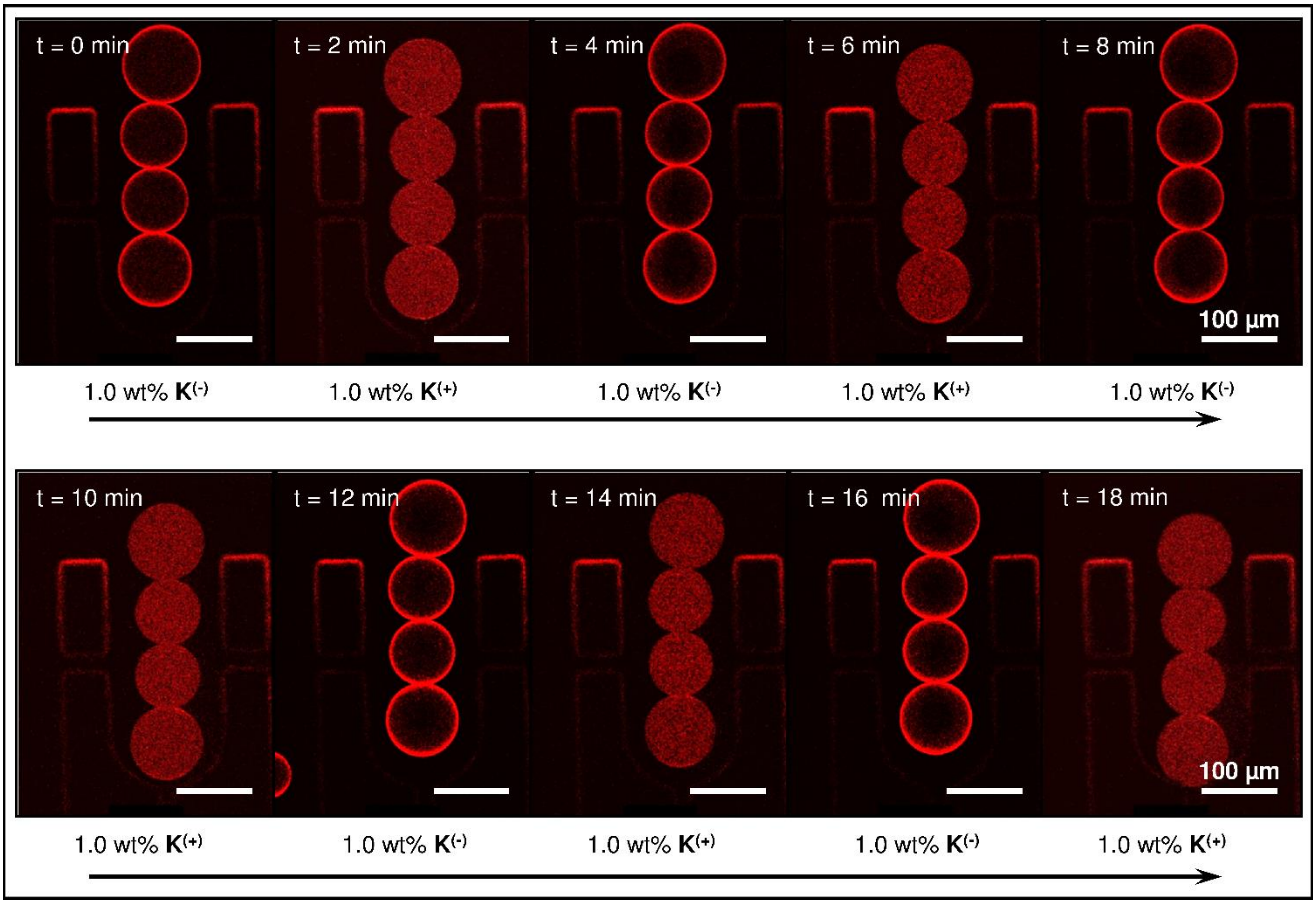


**Figure S10 | Dynamic manipulation of 2B$^{(+)}$ within a microdroplet.** LSCM micrographs of aqueous microdroplets of rhodamine-labelled **2B**$^{(+)}$ [MV = 60 µM], held within the trap by a continuous oil flow (250 µL/h). On alternating between 1.0 wt% of orthogonally charged surfactants **K**$^{(-)}$ and **K**$^{(+)}$ within the carrier oil, **2B**$^{(+)}$ can be repeatedly and controllably assembled at the interface or dispersed throughout the microdroplet, respectively. At 250 µLh$^{-1}$ diffusion to/from the interface was complete within 25 s for all four droplets.

## S4: Supramolecular ternary complex formation with CB[8]

Each cucurbit[8]uril is capable of accommodating two aromatic guests within its cavity to form a 1:2 CB[8]•(guest)$_2$ ternary complex. Figure S11 describes the two-step, three-component formation of the CB[8] ternary complex in water with electron-deficient MV$^{2+}$ (blue) and electron-rich azobenzene (red) guests.

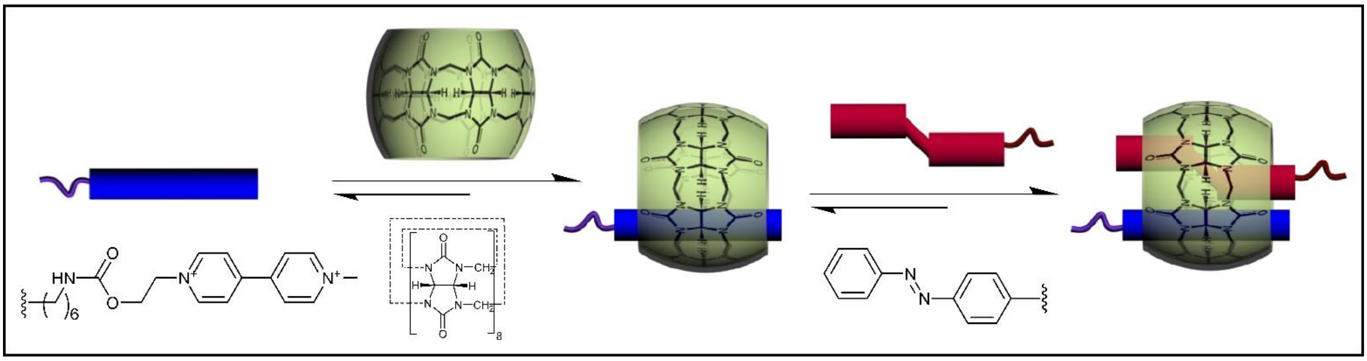

**Figure S11 | The two step mechanism for the formation of the three component supramolecular complex between CB[8] and viologen and azobenzene guests.**

## S5: Core-shell microcapsules

Core-shell microcapsules were prepared containing a dextran cargo (non-fluorescent, 70 kDa, 160 µM), that allowed for osmotic re-inflation of the collapsed, dry structures post-formation. On wetting, the microcapsule immediately began to inflate, with the loss of creases and folds (Figure S12A); further expansion allows the three-dimensional nature of the capsule to be observed (Figure S12B). After 40 minutes in water the core-shell microcapsule had returned to its initial spherical shape, with diameter (Ø = 55 µm) comparable to that seen prior to collapse during evaporative formation. In all cases the '**2A**$^{(+)}$⊂CB[8]⊂**2B**$^{(+)}$' outer shell (red) is clearly visible, encapsulating the '**1A**$^{(-)}$⊂CB[8]⊂**1B**$^{(-)}$' core (green).

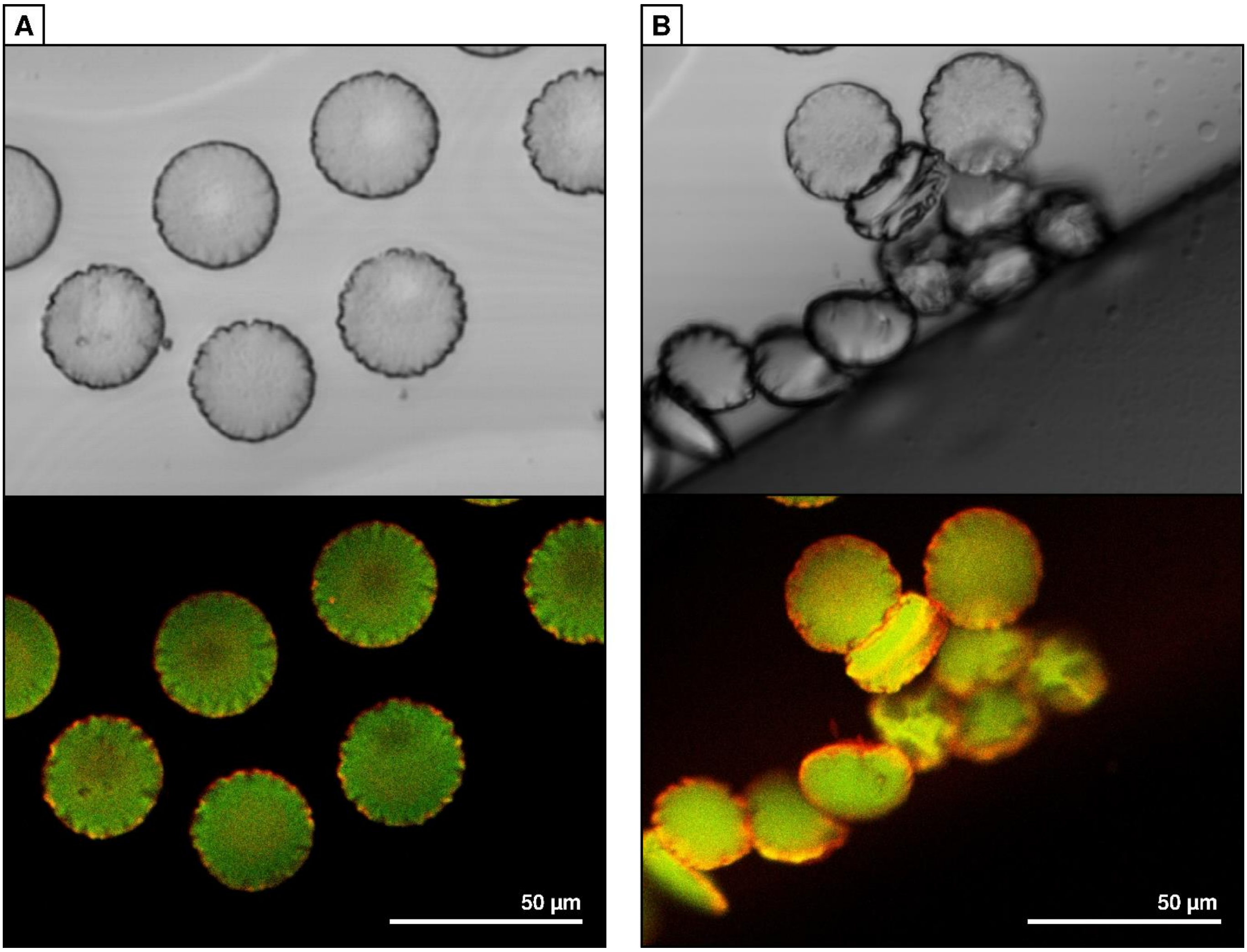


**Figure S12 | Rehydration of core-shell microcapsules.** Transmission and LSCM micrographs of core-shell microcapsules (shell: **2A**$^{(+)}$⊂CB[8]⊂**2B**$^{(+)}$ (red), core: **1A**$^{(-)}$⊂CB[8]⊂**1B**$^{(-)}$ (green)) containing 160 µM of 70 kDa dextran cargo. (**A**) Immediately after wetting the capsule starts to re-inflate, with loss of creases and folds. (**B**) Partially re-inflated microcapsules accumulated at an air/water interface, showing their three-dimensional structure. In all cases the core-shell structure is clearly visible.

## S6: '2A$^{(+)}$⊂CB[8]⊂2B$^{(+)}$' microcapsules formed in hexadecane oil

The electrostatically-directed self-assembly of supramolecular microcapsules is not dependent upon the use of perfluorinated oils in the continuous phase, but simply upon the presence of an appropriately charged surfactant at the interface. This is exemplified in Figure S13, where **2A**$^{(+)}$⊂CB[8]⊂**2B**$^{(+)}$ microcapsules are analogously formed from microdroplets generated with hexadecane carrier oil, with controlled flow rates of 75 and 150 µLh$^{-1}$ respectively. Here Span-80 (2.5 wt%) is used as a surfactant to stabilise the droplets, while palmitic acid (0.5 wt%) is used as a substitute for the negatively-charged perfluorinated dopant, **K**$^{(-)}$. Upon evaporation of the aqueous phase, crumples became apparent on the microcapsule surface, resulting in eventual collapse on loss of the aqueous core. As before, evidence of a polymeric skin around the microdroplet is observed at *circa* 70 % of the initial droplet diameter.

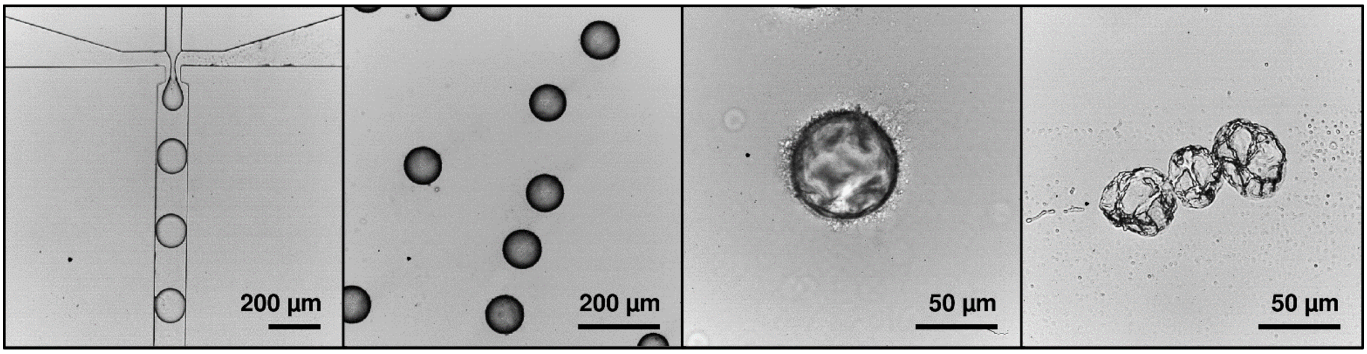


**Figure S13 | $2A^{(+)}$⊂CB[8]⊂$2B^{(+)}$ microcapsules formed in hexadecane oil.** Transmission micrographs of (i) generation of aqueous microdroplets in hexadecane oil containing 2.5 wt% Span-80 surfactant and 0.5 wt% palmitic acid as a negatively-charged dopant; (ii) monodisperse aqueous microdroplets (Ø = 94 µm); (iii) formation of the crumpled microcapsule skin upon evaporation; (iv) dry, collapsed microcapsules.

## S7: Synthetic methods

**Instrumentation.** $^{1}$H NMR spectra (400 MHz) were collected on a Bruker Avance QNP 400 MHz Ultrashield spectrometer, equipped with a 5-mm BBO ATM probe with a *z*-gradient. Weight average molecular weight ($M_W$), number average molecular weight ($M_n$) and polydispersity ($M_W/M_n$) were obtained by aqueous GPC. The aqueous GPC setup consisted of a Shodex OHpak SB column, connected in series with a Shimadzu SPD-M20A prominence diode array detector, a Wyatt DAWN HELEOS multi-angle light scattering detector and a Wyatt Optilab rEX refractive index detector.

**Materials.** All reagents were purchased from Sigma Aldrich, except fluorescein o-methacrylate, which was purchased from Alfa Aesar. [3-(Methacryloylamino)propyl] trimethylammonium chloride solution, sodium 4-vinylbenzenesulfonate, N-hydroxyethyl acrylamide, N,N-dimethylaminoethyl methacrylate and acrylic acid monomers were passed through a column of silica gel and purged with high purity nitrogen for one hour prior to use. Solvents and reagents were used without further purification unless otherwise stated. All aqueous solutions were prepared in deionized water (Millipore Milli-Q Gradient A10) ensuring a resistivity of >15 MΩcm$^{-1}$.

**Preparation of CB[8].** Cucurbit[8]uril was synthesized from glycoluril and formaldehyde, following the procedure described by Day and Kim,[1,2] further isolation and purification followed the previously reported method.[3]

**Preparation of amine-terminated Krytox, $K^{(+)}$.** To a stirred solution of Krytox 157FS-L (16.70 g, 6.91 mmol) in HFE-7100 (20.0 mL) at room temperature, under nitrogen, was added oxalyl chloride (2.41 mL, 27.64 mmol, ca. 4.0 mol eq.). After 5 mins dimethylformamide (1 drop, ca. 10 µL) was added *via* syringe, which initiated effervescence and the solution stirred was for 22 h. The solution was removed by syringe and was evaporated to dryness to give the acid chloride as a clear oil (16.58 g, 98.6%), which was used without further purification. IR (neat oil) *v* = 1808.3 cm$^{-1}$ (s, C=O).

To ethylene diamine (6.69 mL, 0.1 mol), in a 20.0 mL microwave vial with stirring, was added a solution of the acid chloride (4.87 g, ca. 2.00 mmol) in FC-3283 (5.0 mL) dropwise over 15 minutes. The mixture was then heated, with stirring, at 150 °C in a microwave for 30 minutes. The maximum observed pressure was 9 bar at 157 °C. On cooling two layers were observed. To the crude reaction mixture was added (HFE-7100, 6.0 mL) and methanol (6.0 mL) and was rapidly stirred at room temperature for 1H. The stirring was stopped and the two layers allowed to settle, the supernatant was then carefully removed and discarded *via* syringe and methanol (10.0 mL) was added followed by a further 1 h stir, before the methanol supernatant was again removed and discarded. The lower layer was then rapidly stirred with tetrahydrofuran (10.0 mL), after 16 h the supernatant was again carefully removed and discarded. This extraction process was repeated as tetrahydrofuran (10.0 mL) was added and rapidly stirred for 1 h and the tetrahydrofuran supernatant was discarded. Finally the lower layer was diluted with HFE-7100 (12.5 mL), the solution was filtered and evaporated to dryness to give a 1:1, mixture by $^{1}$H NMR of the product (**$K^{(+)}$**) and a tri-block impurity as a clear colorless oil (4.863g, 98.9%). This was used without further purification. IR (neat oil) *v* = 1705 cm$^{-1}$ (s, C=O), 3362.4 cm$^{-1}$ (w, $NH_2$). $^{1}$H NMR (400 MHz, 5% $CDCl_3$ in perfluorooctane): **$K^{(+)}$** [7.58 (1H, bs, N**H**$CH_2CH_2NH_2$), 3.58 (2H, bd, CONHC**$H_2$**, J = 6.0 Hz), 3.08 (2H, t, C**$H_2$**$NH_2$), 1.46 (2H, bs, N**$H_2$**,)], tri-block impurity [7.92 (2H, bs, ($CH_2$N**H**)$_2$,), 3.87 (4H, m, C**$H_2$**NH)$_2$)].

**Preparation of poly(HEAm-*co*-AmAm-*co*-fluor-*co*-azobenzene), $1A^{(-)}$.** The precursor polymer poly(N-hydroxyethyl acrylamide-*co*-(3-acrylamidopropyl)trimethylammonium chloride) was a kind donation from E. Appel, Ratio of HEAm : AmAm = 914: 86.[4] Azobenzene isocyanate was synthesized as previously reported.[5]

A solution of poly(N-hydroxyethyl acrylamide-co-(3-acrylamidopropyl)trimethylammonium chloride) (262 mg) in N-methyl-2-pyrrolidinone (2 mL, anhydrous) was heated to 60 °C for 10 minutes under an inert nitrogen atmosphere. After the polymer solution was cooled to room temperature, fluorescein isothiocyanate (9 mg, 0.023 mmol, 1 mol%) dissolved in NMP (0.5 mL, anhydrous), azobenzene isocyanate (38 mg, 0.17 mmol, 7.5 mol%) dissolved in NMP (0.5 mL, anhydrous) and dibutyltin dilaurate catalyst (1 drop) were added sequentially. The reaction flask was covered with foil and the mixture was stirred for 24 h at room temperature. The orange product was obtained by precipitation from ethyl acetate (3x50 mL). The formed polymer was characterized to give $M_W$ = 121 kDa, $M_n$ = 103 kDa, with PDI = 1.2 from GPC. $^1H$ NMR spectrum in $D_2O$ is shown below:

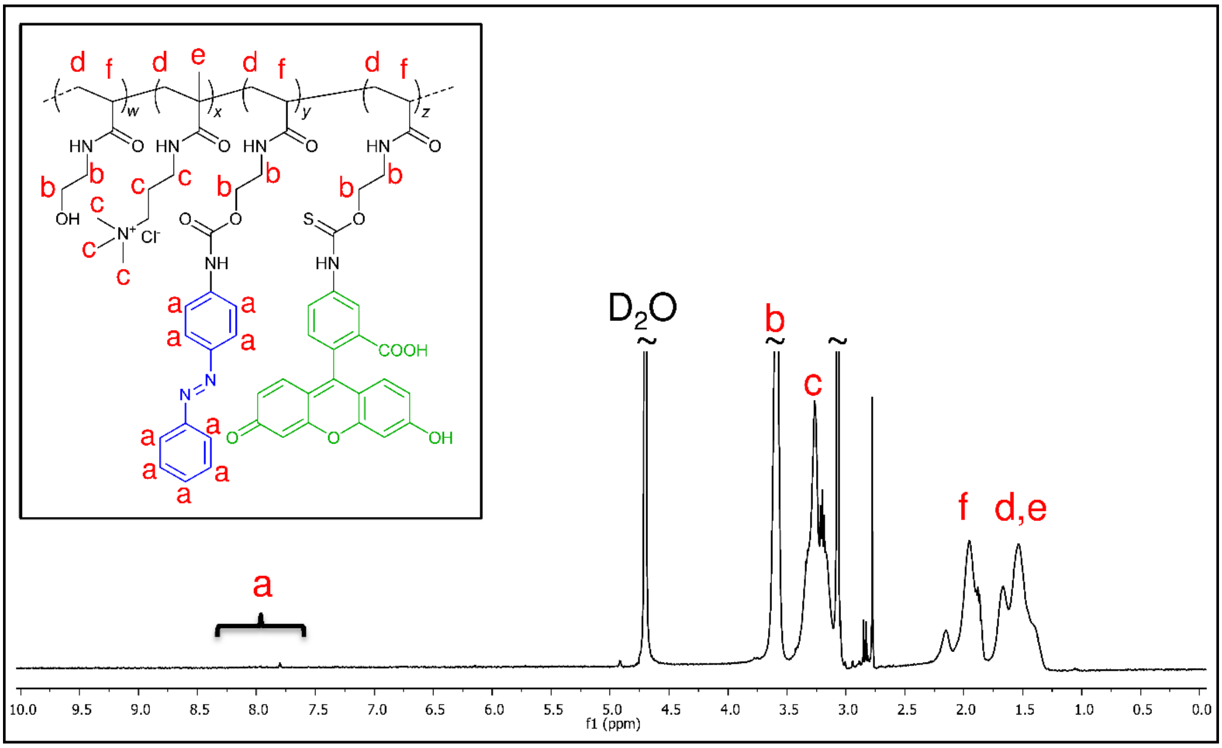


**Preparation of 1-methyl-1'-(4-vinylbenzyl)-[4,4'-bipyridine]-1,1'-diium chloride iodide, MV-styrene.** MV-styrene was synthesized by a one-step electrophilic substitution.[6] Typically, 1-methyl-4,4'-bipyridinium iodide (6.02 g, 20 mmol) was placed in a 500 mL flask with addition of acetonitrile (260 mL). The mixture was heated to 80 °C until all 1-methyl-4,4'-bipyridinium iodide dissolved. To this, 4-cholomethylstyrene (10 mL, 60 mmol) was added and the mixture heated with stirring at 60 °C for 24 hours. After cooling to room temperature, the orange crude product was filtered, washed with acetonitrile and dried under reduced pressure to give a yellow-orange powder (5.4 g, 60%). $^1H$ NMR (500 MHz, $D_2O$), 9.09 (d, 2H), 9.0 (d, 2H), 8.46 (q, 4H), 7.56 (d, 2H), 7.45 (d, 2H), 6.77 (dd, 4H), 5.85 (t, 4H), 5.37 (d, 2H), 4.46 (s, 1H).

**Preparation of poly(AmAm-*co*-SS-*co*-fluor-*co*-StMV), 1B$^{(-)}$.** Poly(AmAm-*co*-SS-*co*-FITC-*co*-StMV) was synthesized *via* conventional free radical polymerisation using 4,4-azobis(4-cyanovaleric acid) as an initiator. To a two-necked round bottom flask was added [3-(methacryloylamino)propyl] trimethylammonium chloride solution (50 wt% in water, 2.21 g, 5.0 mmol, 400 mol eq.), sodium 4-vinylbenzenesulfonate (0.52 g, 2.5 mmol, 200 eq.), MV-styrene (0.11 g, 0.25 mmol, 20 eq.) and fluorescein o-methacrylate (0.06 g, 0.15 mmol, 12 eq.) in aqueous methanol (50 wt%, 4.0 mL). Oxygen was removed by bubbling argon through the solution for 20 mins, followed by the subsequent addition of 4,4-azobis(4-cyanovaleric acid) (3.5 mg, 0.0125 mmol, 1 eq.). The stirred mixture was heated for 24 hours (70 °C, 400 rpm). The resultant polymer was dialyzed in water through a MWCO 6,000-8,000 membrane and freeze dried. The formed, pale yellow polymer was characterized as $M_W$ = 34 kDa, $M_n$ = 28 kDa, with PDI = 1.2 from GPC. A monomer ratio of AmAm: SS: StMV = 13: 11: 1 was calculated from $^1H$ NMR spectrum in $D_2O$, below:

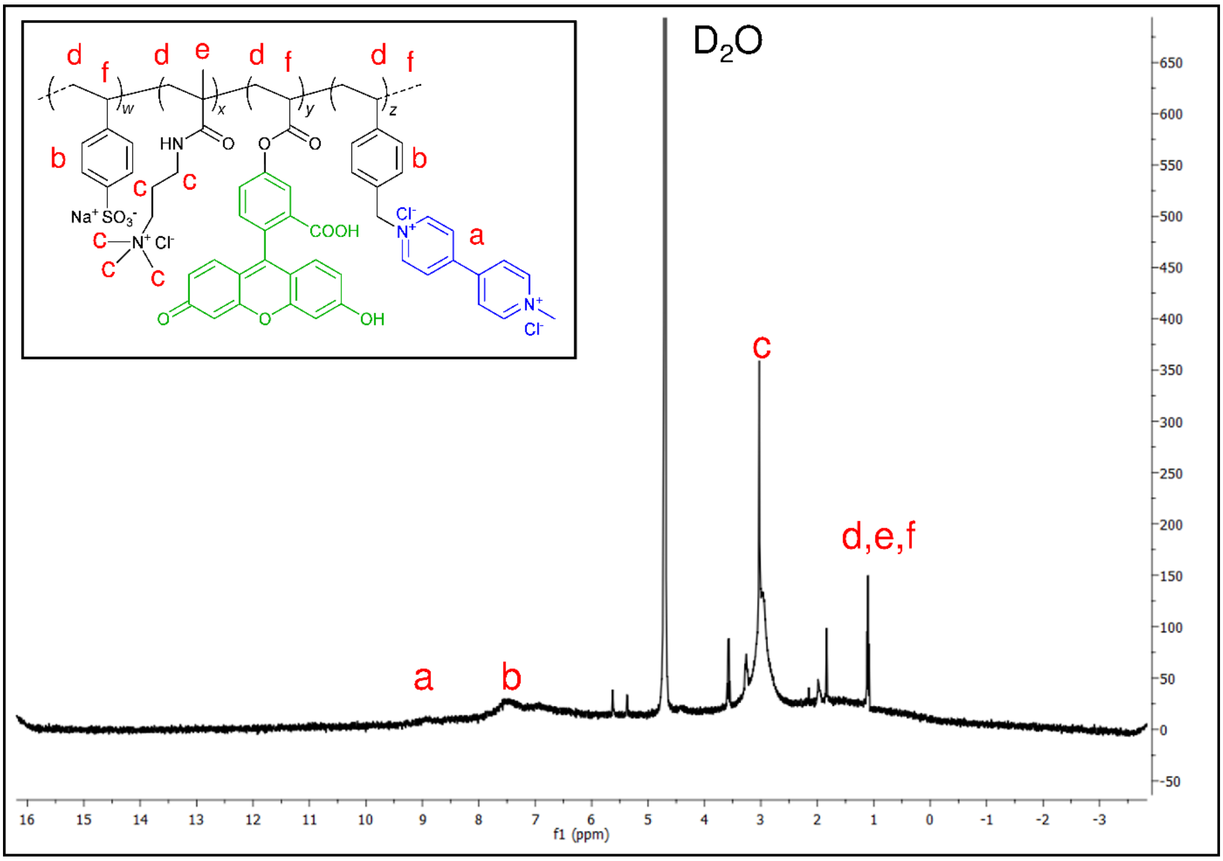


**Preparation of poly(VA-*co*-rhodB-*co*-MV), 2B$^{(+)}$.** MV-Hex-NCO dihexafluorophosphate was prepared as previously reported.[5] To a solution of 1-(2-hydroxyethyl)-1'-methyl-[4,4'-bipyridine]-1,1'-diium di(hexafluorophosphate) (0.5 g, 1.0 mmol) in 200 mL anhydrous acetonitrile was added an excess 1,6-hexamethylene diisocyante (2.0 mL) and a drop of dibutyltin dilaurate. The reaction mixture was then stirred for 24 h at room temperature, before concentrating *in vacuo* to approximately 10 mL, with subsequent addition of anhydrous diethyl ether (200 mL). After 30 mins stored in the freezer, the solvent was decanted and the residue dissolved in the minimum amount of anhydrous acetonitrile. This was repeated four times before drying *in vacuo* to give a yellow, sticky solid was dried under reduced pressure to yield MV-Hex-NCO dihexafluorophosphate (80% yield).

To purify polyvinylalcohol (Mowiol 6-98, Mw-47kDa, 10.0 g), it was dissolved in water (150 mL) and heated (60 °C) for 3 h, before allowing it to settle overnight, decanted and freeze dried. The purified PVA (100 mg) was dissolved in anhydrous N-methyl-2-pyrrolidone (10 mL) and to this were added simultaneously: rhodamine B isothiocyanate (1 mg), MV-Hex-NCO dihexafluorophosphate (127 mg, 10% mol ratio of PVA) and a drop of dibutyltin dilaurate. The mixture was stirred for 24 h at room temperature, dialyzed in water through a MWCO 6,000-8,000 membrane and freeze dried. The formed polymer was red in color and characterized as $M_W$ = 51 kDa, $M_n$ = 28 kDa and PDI = 1.3 from GPC. A monomer ratio of PVA: MV = 18.2: 1 was calculated from $^1$H NMR spectrum in $D_2O$, below:

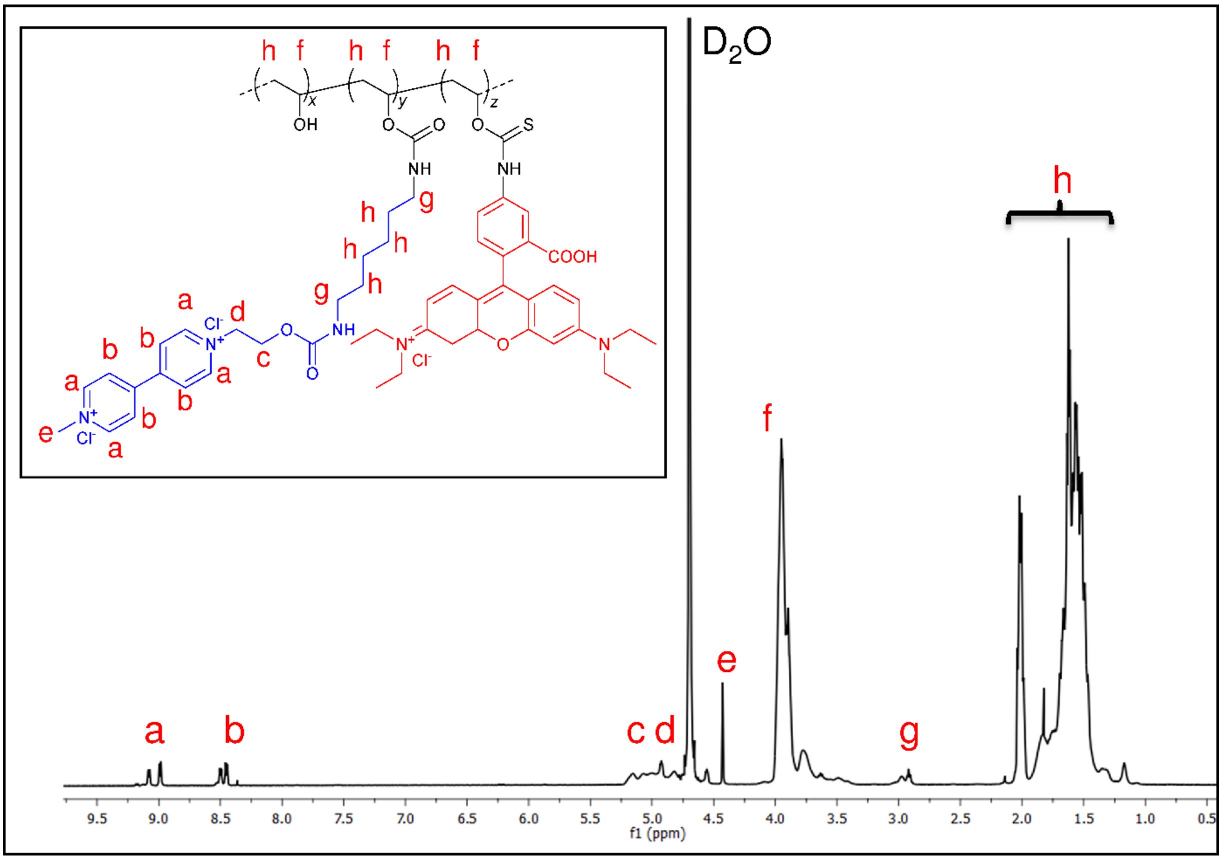

**Preparation of poly(VA-*co*-rhodB-*co*-stilbene), 2A$^{(+)}$.** Stilbene isocyanate was synthesized as previously reported.[5] Poly(VA-*co*-rhodB-*co*-stilbene) was synthesised analogously to **2B$^{(+)}$**, *via* the addition of stilbene isocyanate (10 mol%) and rhodamine B isothiocyanate (1 mol%) to purified polyvinylalcohol (Mowiol 6-98, Mw-47kDa). The formed polymer was red/purple in color and was characterized as $M_W$ = 73 kDa, $M_n$ = 56 kDa and PDI=1.3, from GPC. $^1$H NMR spectrum in $D_2O$ is shown below:

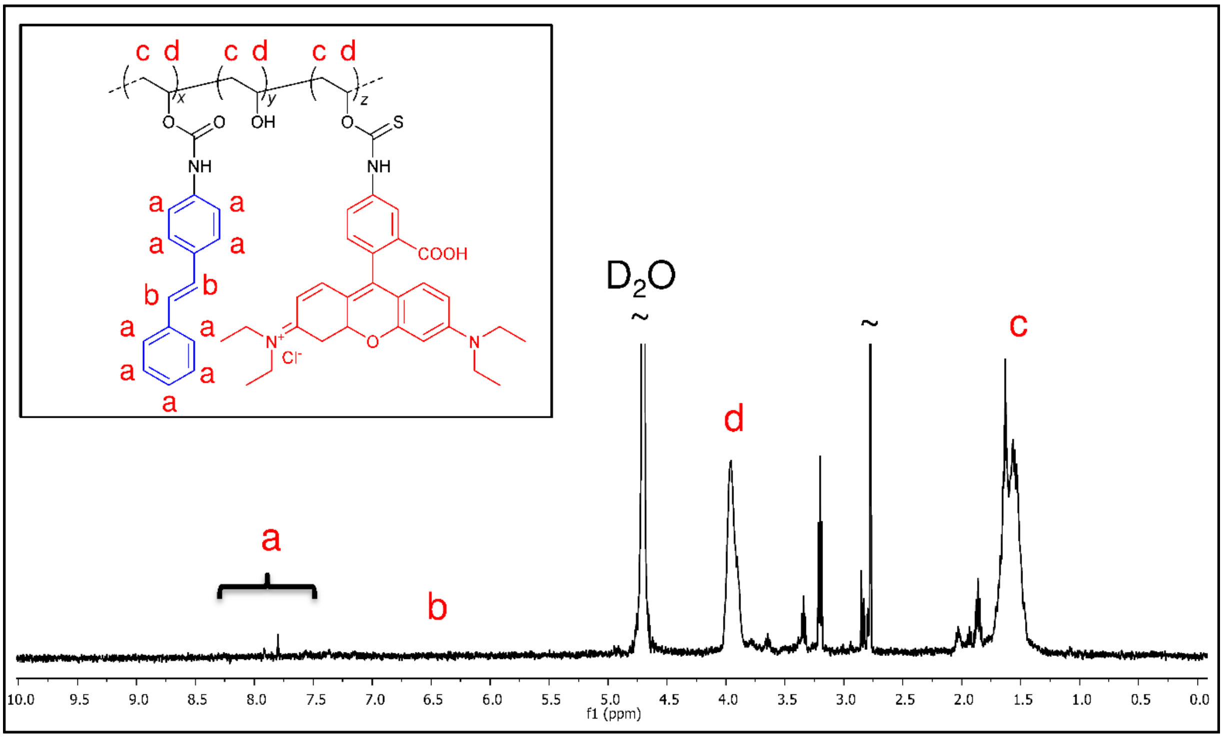

## References


[1] A. Day, A. P. Arnold, R. J. Blanch, B. Snushall, *J. Org. Chem.* **2001**, *66*, 8094.

[2] J. Kim, I.-S. Jung, S.-Y. Kim, E. Lee, J.-K. Kang, S. Sakamoto, K. Yamaguchi, K. Kim, *J. Am. Chem. Soc.* **2000**, *122*, 540.

[3] D. Jiao, N. Zhao, O. A. Scherman, *Chem. Commun.* **2010**, *46*, 2007.

[4] E. A. Appel, F. Biedermann, U. Rauwald, S. T. Jones, J. M. Zayed, O. A. Scherman, *J. Am. Chem. Soc.* **2010**, *132*, 14251.

[5] F. Biedermann, E. A. Appel, J. del Barrio, T. Gruendling, C. Barner-Kowollik, O. A. Scherman, *Macromolecules* **2011**, *44*, 4828.

[6] T. Ogoshi, K. Masuda, T.-A. Yamagishi, Y. Nakamoto, *Macromolecules* **2009**, *42*, 8003.
